# Deciphering the drivers of antibiotic resistance gene transmission in the megacity: Co-occurring contaminants and bacterial community

**DOI:** 10.1016/j.eehl.2026.100242

**Published:** 2026-04-20

**Authors:** Fangfang Ding, Ye Li, Tianhao He, Yuyi Wang, Yushan Li, Ye Huang, Guoyu Yin, Jing Yang, Yuyan Liu, Yan Li, Tao Li, Lijun Hou, Min Liu

**Affiliations:** a Key Laboratory of Geographic Information Science (Ministry of Education), School of Geographic Sciences, East China Normal University, Shanghai 200241, China; b College of Urban and Environmental Sciences, Hubei Normal University, Huangshi 435002, China; c College of Geography and Environmental Science, Hainan Normal University, Haikou 571158, China; d Collaborative Innovation Center of Sustainable Forestry, College of forestry, Nanjing Forestry University, Nanjing 210037, China; e Taihu Basin Shallow Lake Ecosystem Observation and Research Station, Ministry of Water Resources, Wuxi 214024, China; f State Key Laboratory of Estuarine and Coastal Research, East China Normal University, Shanghai 200241, China

**Keywords:** Antibiotic resistance genes (ARGs), Co-occurring contaminants, Land use, Environmental risk assessment, Microplastics, Microbial community, Urban water, Urban sediment

## Abstract

Urban waters are widely contaminated with co-occurring microplastics and antibiotics. Human-land interactions (e.g., wastewater discharge, stormwater runoff, and land use) drive contaminant distribution and antimicrobial resistance. Nevertheless, there is a lack of systematic research evaluating the role of co-occurring contaminants in shaping the spread of antibiotic resistance genes (ARGs). In this study, a metagenomic approach was used to characterize the diversity and distribution of ARGs based on contaminant co-occurring patterns. The random forests and partial least squares path model (PLS-PM) were used to identify and prioritize the factors impacting ARGs, leading to a thorough environmental health ecological risk evaluation. Industrial waters, especially pharmaceutical factories, were significant reservoirs and hotspots for the development of ARGs. Urban estuaries further gathered and amplified the effects of co-occurring contaminants, thereby enhancing the prevalence of ARGs. The potential spread of ARGs was dominated by contaminant co-occurring patterns in urban waters, whereas microbial communities dominated in sediments. Urban zoning comprehensively affected environmental health risks, indicating that environmental management strategies, such as controlling pollution sources and implementing remediation, should prioritize water bodies in agricultural areas and sediments in commercial/residential areas.

## Highlights

• Human activities shaped the spatial distribution patterns of ARGs.
• Co-occurring contaminants in water can track the footprint of ARGs.
• Rubber microparticles in pipelines may serve as hotspots for ARG dissemination.
• Environmental health risk of multiple pollutants was comprehensively assessed.

## Graphical abstract

**Figure:**
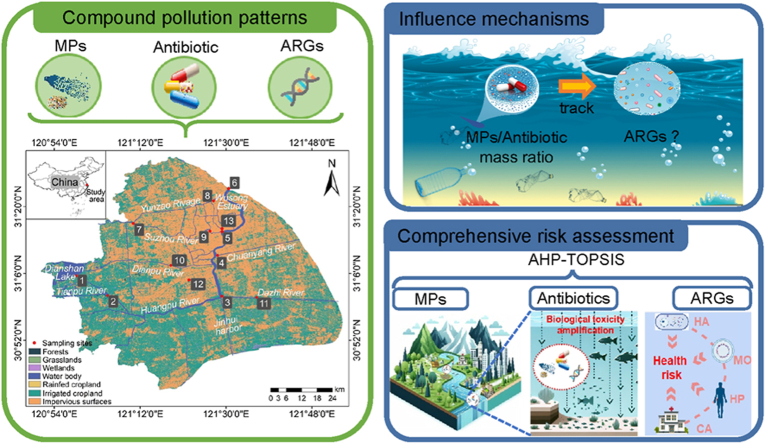

## 1 Introduction

Antimicrobial resistance (AMR), recognized by the World Health Organization as a critical One Health challenge, is exacerbated by the environmental dissemination of antibiotic resistance genes (ARGs)—emerging genetic contaminants widely detected in natural and engineered ecosystems [[1], [2], [3], [4]]. The overuse of antibiotics has accelerated the proliferation of ARGs and their microbial hosts, thereby elevating public health risks through potential transmission via food chains and direct contact [2]. Microplastics (MPs), defined as plastic particles smaller than 5 mm, facilitate pollutant transport primarily through adsorption, a process enhanced by their hydrophobicity, large surface area, and porous structure [5,6]. More importantly, MPs provide a stable substrate for microbial colonization, forming a distinct ecosystem known as the “plastisphere” [[7], [8], [9]]. This niche not only enriches diverse microorganisms but also potentially accelerates the horizontal gene transfer (HGT) of ARGs, as evidenced by the co-localization of ARGs, mobile genetic elements (MGEs), and diverse bacteria on MP surfaces [[10], [11], [12]]. Urban water bodies function as major sinks for multiple pollutants, including MPs, antibiotics, and ARGs [13,14]. These pollutants frequently co-occur due to overlapping sources [14,15]. In this study, co-occurring contaminants refer to the simultaneous presence and interaction of antibiotics and MPs in the environment, which collectively exert combined selective pressures on ecosystems. While the individual roles of MPs and antibiotics in shaping ARG dynamics are partially understood [16,17], their combined impact within realistic urban matrices remains largely unquantified. Critically, the relative contribution of co-occurring contaminants versus microbial communities in driving ARG propagation remains unclear, impeding the development of predictive frameworks and effective risk mitigation strategies.

Shanghai, the central city of the Yangtze River Delta urban agglomeration, is characterized by a high level of urbanization and population density and is located within the river basin exhibiting the highest reported burdens of antibiotic contamination in China [[18], [19], [20]]. Anthropogenic activities, such as urban wastewater effluent and specific land-use practices, create primary selection pressures, while the microbial community composition and physicochemical conditions of the water body mediate the ecological processes of ARG transfer and persistence [19,21]. Source identification, integrating Source Tracker and specific ARG indicators, attributed the highest abundance and diversity of ARGs in the urban system predominantly to wastewater discharges and fecal pollution from human and animal activities [[22], [23], [24]]. Urbanization has enriched resistance genes and facilitated potential MGE-mediated transmission in riverine MPs, and MP polymer composition significantly affected the resistance and transmission risk of resistance genes [25]. Antibiotic concentration was significantly correlated with the abundance of ARGs in water, and antibiotics played a critical role in the spread of ARGs [26]. The distribution of ARGs in urban water was governed primarily by dissolved oxygen (DO), total nitrogen (TN), and Chlorophyta dynamics, with TN alone emerging as the dominant predictor within the sediment compartment [27]. Compared with the relatively complete data on traditional persistent pollutants, there is currently a lack of data on the impact of the spread of resistance genes in different urban functional areas. The intricate web of influencing factors makes elucidating the key drivers of urban ARGs profoundly challenging. It is unclear how characteristic human activities in each area create unique co-occurrence patterns of microplastics and antibiotics, which in turn drive microbial community dynamics in environmental compartments and the plastisphere, ultimately shaping ARG propagation.

The comprehensive environmental risk posed by co-occurring microplastics, antibiotics, and ARGs cannot be adequately evaluated using conventional single-pollutant assessment frameworks. This limitation arises from potential synergistic or additive interactions among pollutants, where ARGs—functioning as transmissible genetic contaminants—propagate through horizontal gene transfer rather than chemical toxicity [28]. Existing methodologies, designed for individual pollutants [[28], [29], [30]], fail to capture these complex interactions and generate incompatible risk metrics that resist unification. It has become a challenging issue to rationally and comprehensively assess the environmental risks of co-occurring contaminants. The analytical hierarchy process (AHP), a technique for order of preference by similarity to an ideal solution (TOPSIS), is widely used in environmental science management and engineering project management [[31], [32], [33]], especially in areas where multi-criteria decision analysis is required. AHP allows for the hierarchical structuring of complex problems and the systematic weighting of criteria based on expert judgment, while TOPSIS facilitates the ranking of pollution scenarios by their proximity to an ideal solution. In this context, the comprehensive risk assessment method based on AHP-TOPSIS provides a logical framework for evaluating complex environmental pollution, considering both qualitative and quantitative factors, and can provide a more comprehensive and robust assessment when dealing with complex environmental systems involving multiple interacting pollutants. Therefore, this study combines the AHP and TOPSIS methods to explore a balanced and accurate environmental composite pollution risk assessment in order to provide a new paradigm for better prediction and assessment of the environmental risks of composite pollution in urban environments.

To address the critical knowledge gaps in mechanistic understanding and integrated risk assessment, this study was designed to answer the following research questions: (1) How do distinct urban functional areas shape the spatial patterns of ARGs? (2) How do co-occurrence patterns of MPs and antibiotics, versus the microbial community, influence the abundance and mobilization (via MGEs) of ARGs in water and sediment? (3) Can a multi-criteria decision analysis framework (AHP-TOPSIS) successfully integrate multiple pollutants (ARGs, MPs, and antibiotics) to produce a spatially explicit assessment of comprehensive environmental health risk? We hypothesize that media heterogeneity and land use jointly shape the spatial patterns of ARGs, and that the microplastic-to-antibiotic mass ratio is a pivotal predictive metric in aquatic environments. To test these hypotheses, we employed an integrated approach—coupling metagenomics with Random Forests and PLS-PM—to map ARG distribution, prioritize driving factors, and ultimately deliver a quantitative risk assessment across urban functional zones.

## 2 Materials and methods

### 2.1 Sample collection

**Fig. 1:**
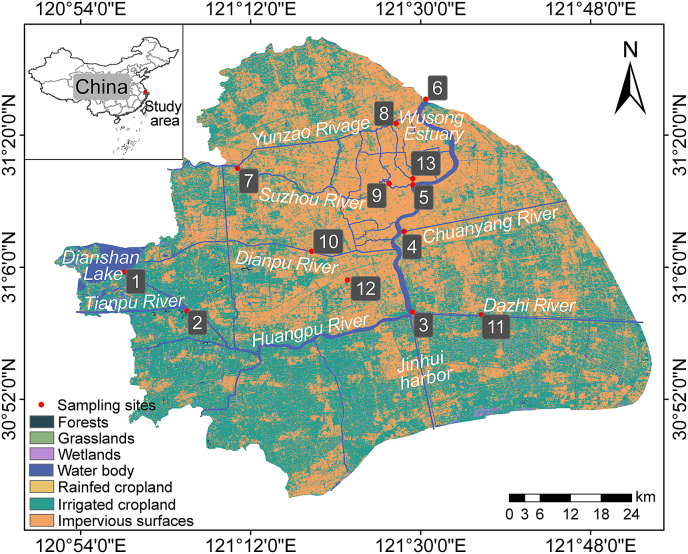
Layout of sampling points within the river network. This base map is based on the standard map GS(2023)2763 obtained from the National Platform for Common Geospatial Information Services (http://bzdt.ch.mnr.gov.cn/).

Based on the land-use type and the surrounding landscape, 13 sampling points in the megacity of Shanghai, China were classified into three urban functional areas (Fig. 1): agricultural areas (site 1, 2, 3, 6, 11), industrial areas (site 4, 8, 12), and commercial/residential areas (site 5, 7, 9, 10, 13) (Fig. S1). Water phase, sediment phase, and particle phase samples were collected at each site. Codes W1−W13 represent sites in the water body, and S1−S13 represent sites in the sediment. The details of the sampling process are presented in Text S1.

### 2.2 DNA extraction and metagenomic analysis

Water samples (1 L) were filtered (0.22 μm), and DNA was extracted in triplicate from the filters and 0.50 g of sediment using the FastDNA Soil Centrifugal Kit. Quality-checked DNA (NanoDrop 2000, gel electrophoresis) was stored at −80 °C. Quality-filtered reads were assembled with Megahit. Open reading frames (ORFs) were predicted from contigs using Prodigal, and genes (≥100 bp) were translated. Cluster Database at High Identity with Tolerance (CD-HIT) was used to create a non-redundant gene catalog (90% similarity). Gene abundance was calculated from SOAPaligner data. Taxonomic/functional annotation was performed via BLASTP against the NR and CARD databases. ARGs and their carrier contigs (ARCs) were identified using DeepARG. ARC abundance was quantified with Burrows–Wheeler Aligner (BWA). The hosts and mobility potential of ARGs were assessed by annotating all ARC ORFs (DIAMOND vs. NR database) and profiling adjacent MGEs. For detailed experimental procedures, please refer to Text S2.

### 2.3 Antibiotics detected by UPLC-MS

Target antibiotic concentrations (Table S1) were quantified using ultra-performance liquid chromatography coupled with tandem mass spectrometry (UPLC-MS/MS; Xevo TQ-S triple quadrupole system, Waters, USA) (Table S1) [34]. More specific details on the extraction method of the antibiotics from environmental samples are provided in Texts S3-S4. Detailed information about the HPLC gradient program for antibiotic separation and the complete MS/MS measurement conditions for individual compounds is presented separately in Table S2. Antibiotics, including sulfonamides (SAs), fluoroquinolones (FQs), tetracyclines (TCs), macrolides (MLs), and chloramphenicols (CPs), were detected. The types of target pollutants are detailed in Text S5.

### 2.4 MPs: Isolation, quantification, and identification

The combination of a stereo microscope (SZX2-ZB16/E3ISPM, Olympus, Japan) coupled with a microscopic Fourier transform infrared spectrometer (Spotlight 400, Perkin Elmer, USA) is a useful tool in order to determine the number of MPs and their characteristics, including size, shape, and color [35,36]. Details of the microplastic detection methods are presented in Text S6. Based on microplastic fragmentation models, the fragmentation coefficient and stability of microplastics in the environment were calculated. For details, please refer to Text S7.

### 2.5 Quality assurance and quality control

The analysis of antibiotics and microplastics was performed to minimize external contamination, and blank and replicate experiments were used to validate the assay and its repeatability. Instruments were calibrated to ensure the reliability of the results. Details on configuring the calibration lines, cleaning the containers, and calculating limits of detection (LODs), limit of quantification (LOQs), and recoveries are presented in Text S8.

### 2.6 The comprehensive environmental health risk

The assessment of risks posed by co-occurring contaminants is a comprehensive and integrative process that requires consideration of both the hazard level and environmental significance of multiple pollutant types (such as microplastics, antibiotics, and ARGs). This involves determining appropriate evaluation criteria and assigning relative weights to individual risk indicators. A hierarchical evaluation model was constructed, with the overall objective being the calculation of a comprehensive hazard index. The model comprises three levels: the goal layer, the criterion layer (microplastics, antibiotics, and ARGs), and the indicator layer. For microplastics, integrated pollution load index (PLI), hazard index (HI), and potential ecological risk index (PERI) were selected [37]. For antibiotics, the risk quotient (RQ) and resistance risk quotient (RRQ) were employed [30]. The health hazard risks of ARGs were evaluated based on four indicators [human accessibility (HA), mobility (MO), human pathogenicity (HP), and clinical availability (CA)] [28]. For ARGs, a risk index (RI) was calculated based on their abundance, mobility, horizontal gene transfer potential, and co-occurrence with antibiotics. For details on the calculation process of multiple indicators, please refer to Texts S9-S11. Subjective weights for each indicator were determined using the AHP, and a pairwise comparison matrix was constructed following Saaty’s 1−9 scale. The relative importance of each indicator was assessed based on its direct and indirect impacts on human health. Matrix consistency was ensured with a consistency ratio (CR) less than 0.1 [32].

Following this, the Positive Ideal Solution (PIS) and Negative Ideal Solution (NIS) were identified, and the Euclidean distances of each sample from these ideal points were calculated. As no NIS was identified in this study, the closeness coefficient (CC) was computed based solely on the distance from the PIS, representing the relative proximity of each sample to the ideal environmental state. The CC was calculated as follows:

(1) $$CC_{i}=\frac{1}{d_{i}^{PIS}}$$

A lower value of $d_{i}^{PIS}$ indicates a shorter distance to the ideal solution, reflecting a higher level of combined pollution risk. Accordingly, samples ranked higher in the TOPSIS results exhibit greater composite pollution hazards. The AHP-TOPSIS approach enables a systematic and robust assessment of the integrated risk posed by microplastics, antibiotics, and antibiotic resistance genes in urban aquatic environments (see Section 4.3). By incorporating both subjective judgments and objective data, this method provides a more comprehensive and reliable evaluation of environmental risks [38].

### 2.7 Statistical analysis

Land-use data at a 30 m resolution were acquired from the Resources and Environmental Sciences Data Platform (RESDC, http://www.resdc.cn). Following the national land-use classification standard [39], seven land-cover categories—forests, grasslands, wetlands, water bodies, rainfed cropland, irrigated cropland, and impervious surfaces—were generated in ArcGIS 10.2 via a supervised maximum-likelihood classification. For each sampling site, landscape metrics, including the comprehensive land-use index (LU), largest patch index (LPI), Shannon’s diversity index (SHDI), and patch density (PD), were extracted using ArcGIS 10.2 and Fragstats 4.0. To ensure spatial comparability, a 500 m-radius buffer was created around every sampling point, and all landscape indices were calculated based on the land-use composition within this standardized buffer zone. Before statistical analysis, the Shapiro-Wilk test was used to assess normality, and Levene’s test was used to evaluate homoscedasticity. The results showed that the data were non-normally distributed and exhibited heteroscedasticity.

Statistical analyses, including the Kruskal-Wallis H test, Wilcoxon rank-sum test, and Spearman correlation, were conducted using SPSS 21.0. The Kruskal-Wallis H test was used to compare the differences in the relative abundance of ARGs across different urban functional zones. The Wilcoxon rank-sum test was applied for pairwise comparisons of these variables between water and sediment compartments. Spearman’s correlation was employed to analyze the correlations between environmental factors (e.g., TN, MPs, and antibiotic concentrations) and ARG abundance. Alpha-diversity, representing the within-sample diversity of ARGs, was calculated using the Shannon index and Simpson’s evenness index. Principal component analysis (PCA) based on Bray-Curtis distances (*p* < 0.05) were calculated in R 4.3.2 using the vegan package. Co-occurrence networks were constructed using Spearman’s correlation (|ρ| > 0.8, *p* < 0.05) and visualized in Gephi 0.10.1. Random forest modeling was conducted in R with the random Forest package after preprocessing the data with dplyr and tidyr. Model hyperparameters, such as ntree, were optimized, and variable importance stability was evaluated through the rfPermute package. The significance of predictors was further verified by computing importance scores from 999 permutations of response variables via the A3 package [40]. Significant environmental predictors identified by the random forest analysis were further analyzed using a partial least squares path model (PLS-PM). The plspm package was used to estimate model reliability, validity, and path coefficients (β), while path diagrams were generated to visualize the connections among latent constructs. A bootstrap procedure was applied to assess the stability of path coefficients, and the Goodness-of-Fit (GOF) index was used to summarize model performance.

## 3 Results

### 3.1 Geographical spatial pattern of ARGs in different environmental media

**Fig. 2:**
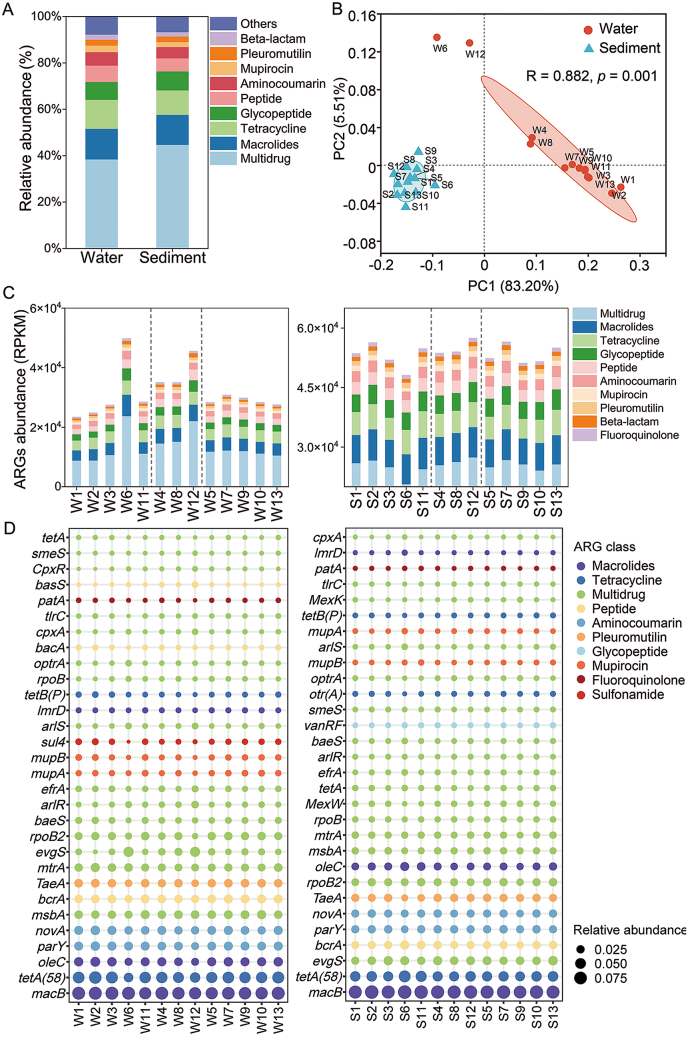
Spatial distribution of ARGs across different functional areas. (A) Composition of ARGs in different environmental media. (B) Principal component analysis (PCA) of ARGs. (C) Abundance of ARGs in water and sediment. (D) Relative abundance of ARGs in different functional areas. Sampling sites categorized by land use type: agricultural areas (sites 1, 2, 3, 6, 11), industrial areas (sites 4, 8, 12), and commercial/residential areas (sites 5, 7, 9, 10, 13). Symbols denote sample matrices: W, water; S, sediment.

The main ARG types in urban water bodies were Multidrug (38.4%), Macrolides (13.1%), Tetracycline (12.5%), Glycopeptide (7.7%), and Peptide (7.0%). The main ARG types in urban sediments were similar to those in water bodies, accounting for 44.7%, 12.9%, 10.6%, 8.2%, and 5.6% of the total abundance in sediments, respectively (Fig. 2A). The average relative abundance of *macB* in sediments was higher than that in water, while the average relative abundance of *tetA(58)*, *parY*, and *oleC* in water was higher than that in sediments (Fig. S2, Text S12). PCA showed that the distribution patterns of ARGs in water and sediment were different, and ARGs in water were affected by point-source pollution and city functional areas, such as W6 and W12 (Fig. 2B and S3). The average abundance of ARGs was higher in sediment (5.65 × 10^4^ RPKM) than in water (3.39 × 10^4^ RPKM). However, the range of ARG abundance was narrower in sediment (5.07 × 10^4^−6.04 × 10^4^ RPKM) compared to water (2.49 × 10^4^−5.34 × 10^4^ RPKM) (Tables S3–S4). The abundance of ARGs in water bodies at W6 and W12 was significantly higher than that in other areas (Fig. 2C).

The top 10 relative abundance ARGs in water environments mainly included Multidrug, Macrolides, Tetracycline, Glycopeptide, Peptide, Aminocoumarin, Mupirocin, Pleuromutilin, Beta-lactam, and Fluoroquinolone (Fig. 2C). The top 30 ARG subtypes relative abundance in the water environment mainly belonged to Multidrug. The relative abundance of *macB* and *tetA (58)* in the water environment was much higher than that of other ARG subtypes. *sul4* was a unique sulfonamide ARG in water, and its abundance varied greatly in urban functional areas. *vanRF* was a unique glycopeptide ARG in sediments, and its abundance did not vary significantly in urban functional areas (Fig. 2D). The relative abundances of *smeS*, *mdtC*, and *mdtB* in the water of industrial areas were significantly higher than those in agricultural areas and commercial/residential areas (Kruskal-Wallis H test, *p* < 0.05) (Fig. S4A). The relative abundance of *PmrF* in sediments of a commercial/residential area was significantly higher than that in the agricultural area and industrial area (Kruskal-Wallis H test, *p* < 0.05) (Fig. S4B).

### 3.2 Multi-contamination in different functional areas

**Fig. 3:**
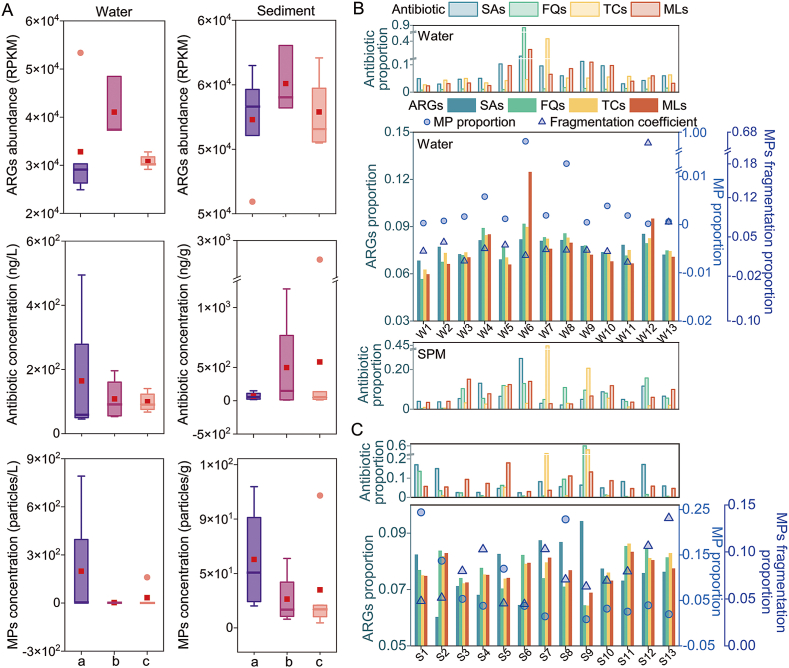
Multi-contamination in different functional areas. (A) ARGs abundance, antibiotics and MPs concentrations in water and sediment across land-use types (a: agricultural areas, b: industrial areas, c: commercial/residential areas). Box plots show the interquartile range (25%−75%), median (line), and mean (square); outliers are indicated by circles. Proportion of antibiotics, ARGs, and MPs in water, suspended particulate matter (SPM) (B), and in sediment (C). SAs, sulfonamides; FQs, fluoroquinolones; TCs, tetracyclines; MLs, macrolides.

In water, ARG abundance showed a trend of industrial area > agricultural area > commercial/residential area, though absolute differences were modest (Fig. 3A, Table S3). This pattern contrasted with the spatial distributions of antibiotics and microplastics. In sediments, ARG abundance was comparable across all functional areas, with the industrial area displaying notably higher variability in its ARG profile (Fig. 3A, Table S4). The variation in ARG abundance in the industrial area was significantly greater compared to the agricultural area and the commercial/residential area.

The concentration ranges of antibiotics in water samples from agriculturalareas, industrial areas, and commercial/residential areas were 45–494, 58–90, and 76–196 ng/L, respectively. The concentration ranges of antibiotics in sediment samples from these areas were 10–149, 20–136, and 13–2680 ng/g, respectively (Fig. 3A). The concentration ranges of MPs in water samples from agricultural areas, industrial areas, and commercial/residential areas were 0.3–791, 0.2–160, and 0.5–4 particles/L, respectively. The concentration ranges of MPs in sediment samples from these areas were 12–117, 18–109, and 4–57 particles/g, respectively (Fig. 3A). The concentrations of MPs in both water and sediment samples from the agricultural area were higher than those in the industrial area and the commercial/residential area.

After normalization of the data for ARGs abundance, antibiotic concentrations, MPs abundance, and fragmentation coefficients, no obvious direct correlation was observed between the same types of antibiotic concentrations and their changes in abundance of ARGs in the water or sediment (Fig. 3B). The abundance of macrolide ARGs in the water was somewhat similar to the changes in the same classes of antibiotics in the water. The abundance of fluoroquinolone ARGs in the water was somewhat similar to the changes in the concentration of FQs in suspended particulate matter. Interestingly, the abundance of ARGs in water and sediment was more intuitively linked to MP abundance and fragmentation coefficients, with MP abundance and fragmentation coefficients combining to influence ARG abundance at different sites (Fig. 3B and C). Site W6 exhibited the highest MP concentration (791.47 particles/L) in water but had a low fragmentation coefficient (0.54), while site W12 showed the most fragmented MP particles (21.26) despite its low MP concentration (0.17 particles/L). At W6, larger particles (>0.1 mm) accounted for 36%, reflecting an unstable fragmentation state, whereas all particles at W12 fell within the 0.025−0.1 mm range, indicating a more stable fragmentation pattern. The corresponding ARGs abundance extremes and hyper extremes were also present at these two sites.

### 3.3 Co-occurrence of host bacteria, MGEs, and ARGs

**Fig. 4:**
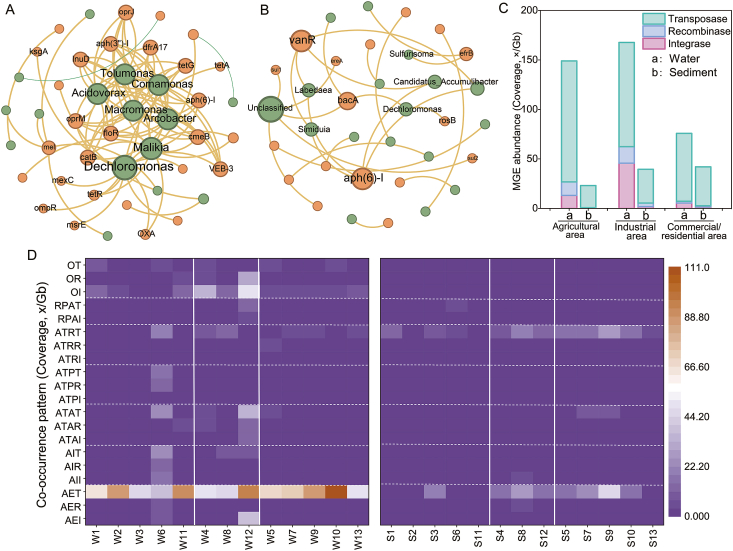
Network correlation analysis between potential host bacteria at the genus level and ARGs in water (A) and sediment (B). Strong correlation represents Spearman’s |ρ| > 0.8 and significant correlation represents *p* < 0.05. The size of the node is proportional to the node degree. (C) MGE concentrations in different functional areas of water and sediment. (D) Abundance of ARCs associated with co-occurrence of ARG resistance mechanism-MGE type. Sampling sites categorized by land use type: agricultural areas (sites 1, 2, 3, 6, 11), industrial areas (sites 4, 8, 12), and commercial/residential areas (sites 5, 7, 9, 10, 13). AEI, antibiotic efflux-integrase; AER, antibiotic efflux-recombinase; AET, antibiotic efflux-transposase; AII, antibiotic inactivation-integrase; AIR, antibiotic inactivation-recombinase; AIT, antibiotic inactivation-transposase; ATAI, antibiotic target alteration-integrase; ATAR, antibiotic target alteration-recombinase; ATAT, antibiotic target alteration-transposase; ATPI, antibiotic target protection-integrase; ATPR, antibiotic target protection-recombinase; ATPT, antibiotic target protection-transposase; ATRI, antibiotic target replacement-integrase; ATRR, antibiotic target replacement-recombinase; ATRT, antibiotic target replacement-transposase; RPAI, reduced permeability to antibiotic-integrase; RPAT, reduced permeability to antibiotic-transposase; OI, others-integrase; OR, others-recombinase; OT, others-transposase.

The co-occurrence pattern between potential host bacteria at the genus level and ARGs in water was more complex than in sediments, likely due to the higher richness and diversity of bacterial communities in water (Text S13, Fig. S5A–C). The water network consisted of 44 nodes and 91 edges. *Dechloromonas*, *Malikia*, *Macromonas*, *Arcobacter*, *Comamonas*, *Acidovorax*, and *Tolumonas* were found to be closely related to various ARG types. *Dechloromonas* was significantly positively correlated with *ksgA*, *ompR*, *oprM*, *OXA*, *tetR*, *msrE*, *tetA*, *VEB-3*, and *mexC*. *Acidovorax* showed significant positive correlations with *oprM*, *cmeB*, *VEB-3*, *lnuD*, *aph(6)-I*, *floR*, *oprJ*, *aph(3″)-I*, *dfrA17*, *tetG*, and *catB*. *Arcobacter* was significantly and positively correlated with resistance genes such as *oprM*, *cmeB*, *VEB-3*, *lnuD*, and *floR* (Fig. 4A). The sediment network consisted of 34 nodes and 26 edges. *Dechloromonas* was significantly positively correlated with *rosB* and *bacA*. *VanR* and *aph(6)-I* were predominantly found in the dominant genera *Candidatus_Accumulibacter*, *Simiduia*, and *Labedaea* (Fig. 4B). The average degree of the network co-occurrence graph between potential host bacteria and ARGs in water (4.14) was higher than that in sediments (1.53). The average path length (2.39) and network density (0.11) of the co-occurrence graph in water were also higher, while its modularity coefficient (0.36) was lower, indicating the robustness of host bacteria in water to the distribution of ARGs.

The average abundance of MGEs in water (142.11 Coverage, ×/Gb) was higher than in sediments (40.43 Coverage, ×/Gb) (Tables S5–S6). The abundance of MGEs in water across different functional areas ranked as follows: industrial area (209.59 Coverage, ×/Gb) > agricultural area (149.96 Coverage, ×/Gb) > commercial/residential area (94.75 Coverage, ×/Gb). In contrast, the abundance of MGEs in sediments ranked in the order of commercial/residential area (52.41 Coverage, ×/Gb) > industrial area (49.35 Coverage, ×/Gb) > agricultural area (23.11 Coverage, ×/Gb) (Fig. 4C, Tables S5–S6). This indicated that, compared to sediments, water bodies had a higher potential for ARG mobility, with ARGs in the industrial area exhibiting stronger diffusion abilities, while ARGs in sediments of the commercial/residential area posed greater diffusion risks. Based on the ARC abundance of different ARG resistance mechanism-MGE type co-occurrence patterns, Fig. 4D shows that ARC abundance in the antibiotic efflux-transposase and antibiotic target replacement-transposase co-occurrence patterns in different media was relatively high, with hotspot values observed in the antibiotic efflux-transposase co-occurrence pattern in water at W10 and sediment at S9.

### 3.4 The underlying factors affecting ARGs propagation

The propagation of ARGs is influenced by various factors, including land use, water quality parameters, co-occurring contaminants, and microbial communities [17,23,41]. The significance of these factors was ranked and predicted using random forest analysis. The results indicated that microbial communities [e.g., relative abundance of species (NR-read-r)], particulate sulfonamide antibiotics (e.g., P-SAs), ARG hosts at the genus level (e.g., Host-Genus-abundance), mobile genetic element (e.g., MGEs-abundance), and multi-contamination [e.g., antibiotic-microplastic mass ratio such as the mass ratio of microplastics to particle-phase antibiotics (MPs-P-AT), the mass ratio of microplastics to particle-phase macrolides (MPs-P-MLs)] were the most robust predictors of ARGs abundance in water (*p* < 0.05) (Fig. S6). In contrast, ARG diversity in water was significantly predicted by water quality parameters (e.g., DO), land use type (e.g., SHDI), and antibiotics in water [e.g., water-phase macrolides (W-MLs)] (*p* < 0.05) (Fig. S6A–B). Additionally, antibiotics in suspended particulate matter [e.g., particle-phase macrolides (P-MLs), particle-phase chloramphenicols (P-CPs)] and sediment [e.g., sediment-phase fluoroquinolones (S-FQs), sediment-phase macrolides (S-MLs)], multiple contaminants [e.g., the mass ratio of microplastics to particle-phase tetracyclines (MPs-P-TCs), the mass ratio of microplastics to sediment-phase sulfonamides (MPs-S-SAs)], ARG hosts at the genus level (e.g., Host-Genus-abundance), microbial community diversity (e.g., NR-simpson), and physicochemical parameters [e.g., total carbon (TC)] were significant predictors of ARGs abundance and diversity in sediments (*p* < 0.05) (Fig. S6C–D).

**Fig. 5:**
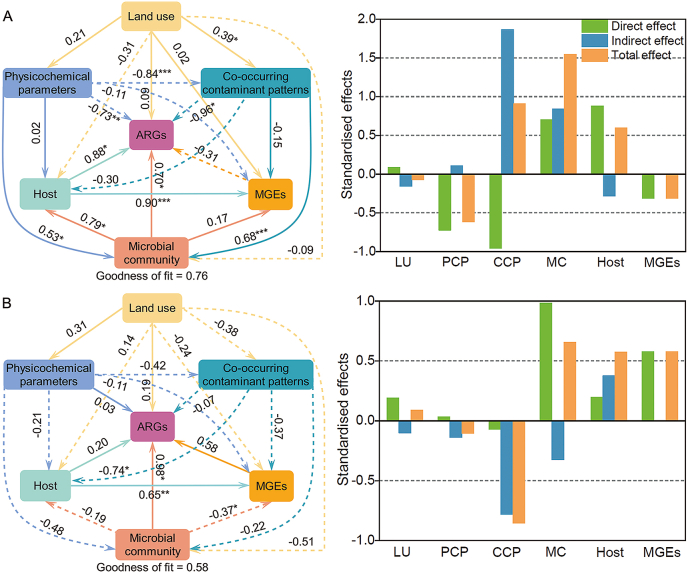
Partial least squares path model (PLS-PM) showing direct and indirect effects of main factors on ARGs in water (A) and sediment (B). The dashed and solid lines represent positive and negative effects, respectively. ∗, ∗∗ and ∗∗∗ represent significance levels of *p* < 0.05, *p* < 0.01 and *p* < 0.001, respectively. LU, land use; PCP, physico-chemical parameters; CCP, co-occurring contaminant patterns; MC, microbial community.

PLS-PM, combined with random forest analysis, was used to elucidate the relationships between numerous complex factors and ARGs. The key indicators revealed by random forest analysis include land use, physicochemical parameters, co-occurring contaminants (e.g., the mass ratio of microplastics to antibiotics in the same volume or mass), microbial communities, and others. In terms of standardized total effects, the most significant factor influencing the abundance of ARGs in water was microbial communities (1.54), followed by the compound pollutant pattern (0.91), physicochemical parameter (−0.62), host bacteria (0.59), MGEs (−0.31), and land use (−0.07) (Fig. 5A). Co-occurring contaminant patterns also significantly affected microbial communities (β = 0.68, *p* < 0.001) and ARGs abundance (β = −0.96, *p* < 0.05). Co-occurring contaminants had the greatest direct impact on the distribution of ARGs in the water, indicating a strong overall impact. Additionally, microbial communities were significantly correlated with ARG hosts at the genus level (β = 0.79, *p* < 0.05) and ARGs abundance (β = 0.70, *p* < 0.05), while ARG hosts were significantly associated with MGEs (β = 0.90, *p* < 0.001) and ARGs abundance (β = 0.88, *p* < 0.05). Physicochemical parameters in water were found to significantly influence co-occurring contaminant patterns (β = −0.84, *p* < 0.001), microbial communities (β = 0.53, *p* < 0.05), and ARGs abundance (β = −0.73, *p* < 0.01). A significant positive correlation was observed between land use intensity in water and the co-occurring contaminant patterns (β = 0.39, *p* < 0.05) (Fig. 5A). In terms of standardized total effects, the most significant factor influencing ARG abundance in sediment was the co-occurring contaminant patterns (−0.85), followed by microbial communities (0.65), MGEs (0.58), host bacteria (0.57), physicochemical parameters (−0.10), and land use (0.09) (Fig. 5B). Microbial communities significantly affected MGEs (β = −0.37, *p* < 0.05) and ARGs (β = 0.98, *p* < 0.05). In sediments, co-occurring contaminant patterns were significantly correlated with ARG hosts at the genus level (β = −0.74, *p* < 0.05), and ARG hosts significantly influenced MGEs (β = 0.65, *p* < 0.01), though the effect of MGEs on ARGs was not significant (Fig. 5B). The results of random forest and PLS-PM suggested that microbial communities and co-occurring contaminants play a potentially dominant role in shaping the spatial distribution of ARGs.

### 3.5 Comprehensive environmental health risk assessment

**Fig. 6:**
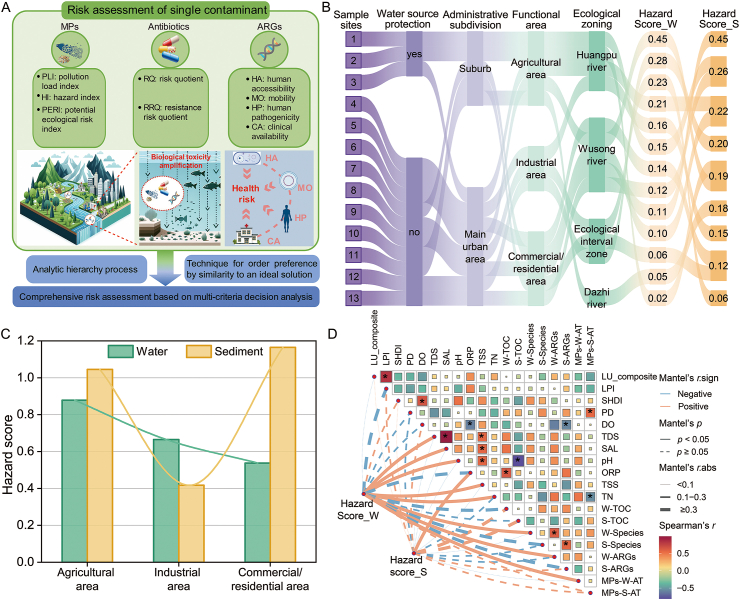
Comprehensive environmental health risk assessment. (A) Main process of comprehensive environmental health risk assessment. (B) Risk levels of co-occurring contaminants in different urban areas. (C) Hazard levels of co-occurring contaminants in different functional areas. (D) Correlation between hazard scores and land use, environmental factors, and co-occurring contaminants. An asterisk indicates significance, and the size of the square indicates the degree of correlation. LU_composite, comprehensive land use index; LPI, largest patch index; SHDI, Shannon’s diversity index; PD, patch density; DO, dissolved oxygen; TDS, total dissolved solid; SAL, salinity; ORP, oxidation-reduction potential; TSS, total suspended solids; TN, total nitrogen; W-TOC, total organic carbon in water; S-TOC, total organic carbon in sediment; MPs-W-AT, the mass ratio of microplastics to water-phase antibiotics; MPs-S-AT, the mass ratio of microplastics to sediment-phase antibiotics.

The urban water environment serves as a primary sink for pollutants generated by industrial and residential activities. This leads to the coexistence and interaction of numerous contaminants, posing significant threats to both the ecological environment and human health. The RQ, RRQ, and HQ of antibiotics were calculated using the predicted no effect concentration (PNEC), the resistance-based predicted no effect concentration (PNEC_R_), and microbiological acceptable daily intakes (ADI_mic_) (Text S9). The hazard risk of microplastics was assessed using indicators such as PLI, HI, and PERI, all based on the abundance and polymer types of microplastics (Text S10). The health risk associated with resistance genes was evaluated by considering HA, MO, HP, antibiotic consumption, and CA (Text S11). The AHP was employed to determine the weights of multiple risk indicators of co-occurring contaminants [31,32], and the TOPSIS was applied for a comprehensive evaluation (Fig. 6A). The comprehensive health risks of multiple pollutants in estuary water were significantly higher than those in urban rivers, whereas the health risks of multiple pollutants in sediments were at a moderate level. Notably, the highest health risks of co-occurring contaminants in urban river water were observed near pharmaceutical factories, and the risks in sediments from commercial, residential, and agricultural areas should not be overlooked (Fig. 6B). The comprehensive environmental health risk levels in sediments from the agricultural area and the commercial/residential area were found to be higher than those in water. Specifically, the risk levels in sediments followed the order: commercial/residential area > agricultural area > industrial area; while in water, the order was: agriculturalarea > industrial area > commercial/residential area (Fig. 6C). The comprehensive environmental health risk of water was significantly positively correlated with water quality parameters, microbial communities, and co-occurring contaminant patterns (*p* < 0.05). The comprehensive environmental health risk of sediments was significantly positively correlated with redox levels [oxidation-reduction potential (ORP)] (Fig. 6D).

## 4 Discussions

### 4.1 Role of media heterogeneity and human activities

The abundance and diversity of ARGs exhibited pronounced heterogeneity across different environmental media in urban rivers, with significantly higher levels in sediments than in water. On the one hand, sediments were more stable than water bodies, with less variation in their physical structure and chemical environment, which allowed ARGs to accumulate in sediments over time [42]. In contrast, ARGs in the water were more susceptible to factors such as water flow, temperature changes, and biological feeding, resulting in relatively low abundance and diversity in the water (Fig. S7). On the other hand, microbial community structures in sediments were typically more complex and diverse than those in the water (Fig. S5). Many pollutants (e.g., antibiotics, heavy metals) in urban streams settle and accumulate in sediments with water flow [43]. These pollutants could not only directly contribute to the production and transport of ARGs but also exert selective pressure on microbial communities.

Urban land use serves multifaceted roles in shaping ARG distribution in the urban river system. Our study identified urban functional areas based on land use and industrial structure. The highest ARG abundance was observed in water from agricultural areas and in sediments from industrial areas (Fig. 2C). Notably, the abundance and diversity of ARGs in water at W6 were significantly different from those in other agricultural areas (W1, W2, W3, and W11). W6 is situated at the confluence of the Huangpu River (an urban river) and the Yangtze River (the longest river in Asia), both of which flow through multiple highly urbanized regions [44], where river confluences contribute to the elevated abundance of ARGs. W12, located near a pharmaceutical factory, might exhibit an elevated abundance of ARGs due to industrial wastewater. The distinct microbial communities at W6 and W12 were critical for the enrichment of ARGs (Fig. S5D–E). In sediments, the most dominant ARG subtypes (e.g., *macB* and *evgS*) may be favored under favorable conditions like low light and nutrient richness [45], which support bacterial survival. In industrial areas, where pollutants and antimicrobial agents were more prevalent, bacteria carrying these resistance genes gained a significant survival advantage (Fig. S4A). In sediments, ARG profiles were predominantly shaped by the native bacterial community. This finding underscores the limitations of end-of-pipe solutions and highlights the need for source control strategies that mitigate the initial input of contaminants, which disrupt microbial ecosystems. Urban planning can mitigate ARG spread by implementing green infrastructure (e.g., constructed wetlands, bioretention basins) in commercial/residential areas to intercept stormwater runoff, and by enforcing stricter pre-treatment standards for industrial wastewater, especially from pharmaceutical and chemical plants. Overall, media heterogeneity and human activities jointly shape the spatial distribution of antibiotic resistance.

### 4.2 The critical effect of co-occurring contaminants

This study suggests that the key factors influencing ARGs dissemination include land use, antibiotic usage, microbial community composition, and horizontal gene transfer (Fig. S6). These factors facilitate the dissemination of ARGs in the environment, complicating efforts to manage and control antibiotic resistance [19,41,46]. The land use, sewage treatment, and discharge could affect the distribution and enrichment of ARGs [23]. Antibiotic residues in the environment contribute to the diffusion and persistence of ARGs by exerting selective pressure, promoting horizontal gene transfer, inducing resistance gene expression, encouraging mutations, and increasing biofilm formation [47]. Microplastics serve as matrices for microbial attachment, adsorb pollutants, alter microbial community structures, and promote the accumulation of resistance genes [16,48]. These mechanisms were inherently coupled, which may obscure direct correlations between ARGs and individual pollutants (e.g., antibiotics, microplastics) (Fig. 3).

The co-occurrence analysis of host bacteria, MGEs, and ARGs in the two environmental media revealed that host bacteria in the aqueous phase were more likely to harbor ARGs and acquire MGEs (Fig. 4). Bacteria belonging to genera such as *Dechloromonas*, *Comamonas*, and *Acidovorax* exhibit broad metabolic capabilities, enabling them to degrade a wide range of pollutants, including organic compounds and heavy metals [49,50]. Additionally, through the degradation of pollutants [51], these bacteria may acquire ARGs from other species via HGT. In sewage treatment plants, species like *Dechloromonas* and *Comamonas* frequently become dominant due to their adaptability and metabolic diversity [50]. A similar pattern was observed in the host bacteria of ARGs in this study (Fig. 4A), highlighting the significant role of municipal wastewater in ARG enrichment. High antibiotic concentrations in these effluents, along with other selective pressures, promote the enrichment of ARGs within these genera. In this context, the co-occurrence pattern of multidrug efflux pump resistance and transposases played a crucial role in bacterial survival and the propagation of resistance (Fig. 4D).

Results from random forest and PLS-PM analyses indicate that co-occurring contaminant patterns were strongly associated with ARG distribution in water, likely contributing to their spread by creating synergistic selection pressures (Fig. 5). The co-occurring contaminant pattern was primarily explained and predicted by the concentration of antibiotics in water and suspended particles, the number and size diversity of microplastics, and the mass ratio of microplastics to antibiotics. Among these observed variables, the mass ratio of microplastics to antibiotics exhibited the highest explanatory power (loading >0.95). This finding suggests that lower microplastic mass per unit volume, combined with larger antibiotic masses, facilitates the enrichment of ARGs. Microplastic particles, being small with a large specific surface area, are prone to adsorb antibiotics and harbor microorganisms, thereby forming a stable microenvironment, such as a biofilm. Biofilms are ideal environments for gene exchange, including HGT, which promotes the enrichment of ARGs [8]. In this study, the abundance of ARGs in water samples W6 and W12 was significantly higher than at other sites (Fig. 2B and C). Interestingly, the microplastic communities in these two locations exhibited distinct characteristics. The proportion of microplastic particles less than 0.1 mm at W6 and W12 was higher than 60% (Fig. S8). The microplastic polymers at W6 were mainly rubber type 3, while at W12, polystyrene was predominant (Fig. S9). The adsorption sites of these microplastic polymers mainly rely on van der Waals forces and π−π interactions [52], making them more likely to adsorb aromatic compounds or other nonpolar organic molecules. Our analysis identified rubber (polymer type 3) as a significant microplastic category in the urban water system. Characterized by elastic properties and surface roughness that may enhance pore structures, rubber particles provide abundant molecular adsorption sites [53]. The specific polymer signature corresponds to common plumbing materials (e.g., Gardena 1124, Vaillant DN 63, and FVV), indicating that urban plumbing infrastructure may be a potential source of these rubber microplastics. Given their prevalence and adsorptive properties, these particles may function as important vectors for the concentration and transport of co-pollutants, thereby contributing to ARG persistence and spread. This identified link between infrastructure-derived microplastics and contaminant transport warrants further investigation. The greater the mass of antibiotics, particularly macromolecular antibiotics, the slower their degradation in the environment due to their complex structures [54]. This slow degradation exerts prolonged and strong selection pressure on microbial communities. Bacteria carrying resistance genes are more likely to survive in this high-stress environment, further spreading ARGs. Macrolide antibiotics, in particular, have a significantly higher molecular weight and a more complex structure compared to other antibiotic types [55]. The mass ratio of microplastics to suspended particulate macrolide antibiotics in water was the most significant (*p* < 0.01) (Fig. S6A). Dissolved oxygen (DO) is a key explanatory variable among physicochemical parameters [56], as it influences biofilm formation and structure. DO also affects the redox state of the environment [57], which in turn impacts the persistence of resistance genes and the stability of MGEs, such as plasmids and transposons. Hypoxic environments may increase the stability and transfer frequency of ARGs. Under low DO conditions, some bacteria may enhance the frequency of HGT, as gene transfer under these conditions provides microorganisms with a survival advantage in adverse environments. While co-occurring contaminant patterns in sediments did not show direct effects on ARGs, they significantly influenced host bacteria, which indirectly affected ARGs (Fig. 5B). Microbial communities in different media had direct or indirect positive effects on the spatial patterns of ARGs by altering community structure or facilitating HGT. In summary, the mass ratio of microplastics to antibiotics provided a more robust and integrative predictor of ARG hotspot formation than individual pollutant concentrations. This ratio effectively captures the synergistic selection landscape that drives the spatial epidemiology of antimicrobial resistance in urban watersheds, offering a valuable metric for future risk assessment and source prioritization.

### 4.3 Health threats of co-occurring contaminants in different functional areas

Urbanization and industrialization have significantly impacted water bodies through the introduction of co-occurring contaminants [11,28,29], posing serious risks to ecosystems and human health. However, previous studies have primarily focused on the environmental health risks associated with individual pollutants [28,30,37], neglecting the risks posed by multiple pollutants, especially those arising from different types of pollution (e.g., physical, chemical, and biological). This study provides a novel integrated framework for quantifying the comprehensive risk to urban water environments by simultaneously incorporating multiple critical dimensions, including the material risk of microplastics, the ecological risk of antibiotics, and the clinical availability, human pathogenicity, accessibility, and mobility of ARGs (Fig. 6A). In general, the environmental health risks of co-occurring contaminants were found to be higher in sediments than in water (Fig. 6B), primarily due to the strong adsorption and accumulation capabilities of sediments. Pollutants are highly concentrated in sediments and exhibit strong bioavailability [58]. Moreover, pollutants in sediments are difficult to mitigate and remove, posing a persistent threat to the environment.

Among the different functional areas, the sediment in commercial/residential areas exhibited significantly higher risk levels than those in agricultural areas and industrial areas (Fig. 6C). The dense population and high wastewater discharge characteristic of these zones resulted in inputs of antibiotics and microplastics, whose combined effect—quantified by their mass ratio—was identified as a key driver of ARG abundance. Rainfall runoff from impervious surfaces carries large amounts of particulate pollutants into water bodies, where they rapidly settle [59]. Consequently, sediment in these areas presented a higher risk of co-occurring contaminants. Water hazard risk in agricultural areas was higher than in commercial/residential, and industrial areas (Fig. 6C). This was primarily because agricultural areas often lack centralized pollution control measures but possess extensive irrigation and drainage systems [60]. Runoff from farmland, containing high concentrations of nutrients such as nitrogen and phosphorus, as well as dissolved pollutants like pesticides, fertilizers, and antibiotics, is discharged directly into water bodies [60,61], thereby maintaining high pollutant concentrations. Industrial areas had relatively low levels of sediment pollution due to the effectiveness of centralized treatment measures. We further examined external factors contributing to comprehensive environmental health risks and considered various aspects of urban regional planning. Notably, water environment risks were lower in water source protection areas, while higher risks were observed in water bodies adjacent to the main urban area. The Wusong River exhibited higher sediment environmental risks (Fig. 6B). Urban land use drives contaminant co-occurring patterns that significantly influence integrated human health and ecological risks.

This study systematically addressed the research questions and validated the proposed hypotheses. First, by mapping ARGs across urban functional areas, we demonstrated a spatial pattern: industrial areas were key reservoirs for ARGs. Second, co-occurring contaminants, particularly the microplastic-to-antibiotic mass ratio, were the dominant driver in water, directly influencing ARG abundance. In contrast, the microbial community was the primary regulator in sediments, supporting the hypotheses. Third, the AHP-TOPSIS framework successfully integrated the risks from MPs, antibiotics, and ARGs, generating a spatially explicit, composite risk index that prioritizes zones for intervention. Therefore, this study elucidates the environmental drivers of urban ARG propagation, establishes a predictive mass-ratio metric of pollutants, and delivers an integrated risk assessment framework to guide targeted management.

## 5 Conclusions

Urban sediments harbor a broader diversity of ARGs than overlying waters, largely due to their capacity to support more complex and stable microbial communities. Urban water bodies adjacent to industrial areas, particularly pharmaceutical factories, function as critical reservoirs and hotspots for ARG development, while urban estuaries further accumulate and amplify the impacts of co-occurring contaminants. Integrating PLS-PM modeling with random forest analysis revealed that contaminant co-occurring patterns primarily drive ARG dissemination in urban waters, whereas microbial community structure plays a dominant role in sediments. Microplastic particles in water, especially those with small sizes and porous surface structures (e.g., rubber particles), provide a favorable environment for the adsorption and development of antibiotics and ARGs, facilitating their long-distance transportation. Urban zoning substantially influences environmental health risks, indicating that water bodies in agricultural areas and sediments in commercial/residential areas warrant prioritized management. Environmental authorities should implement a media heterogeneity management strategy that incorporates the microplastic-to-antibiotic mass ratio as a key monitoring metric for water bodies. Targeted source-control strategies, including advanced industrial wastewater treatment and enhanced stormwater filtration in densely populated areas, are essential to mitigate the environmental dissemination of ARGs and associated health risks in urban settings.

## CRediT authorship contribution statement

**Fangfang Ding:** Writing – review & editing, Writing – original draft, Formal analysis, Data curation, Conceptualization. **Ye Li:** Writing – review & editing, Writing – original draft, Formal analysis, Data curation, Conceptualization. **Tianhao He:** Methodology, Formal analysis. **Yuyi Wang:** Visualization. **Yushan Li:** Formal analysis. **Ye Huang:** Conceptualization. **Guoyu Yin:** Data curation. **Jing Yang:** Data curation. **Yuyan Liu:** Validation. **Yan Li:** Investigation. **Tao Li:** Validation. **Lijun Hou:** Conceptualization. **Min Liu:** Writing – review & editing, Funding acquisition, Data curation, Conceptualization.

## Declaration of competing interest

The authors declare that they have no known competing financial interests or personal relationships that could have appeared to influence the work reported in this paper.
