# Supplementary material for: Deciphering the drivers of antibiotic resistance gene transmission in the megacity: Co-occurring contaminants and bacterial community
Source: Eco Environ Health. 2026 Apr 20;5(2):100242. doi: 10.1016/j.eehl.2026.100242 (PMC13213758; doi:10.1016/j.eehl.2026.100242)
Supplement: Multimedia component 1 [file mmc1.docx]

**Supplementary Information for**

**Deciphering the drivers of antibiotic resistance genes transmission in the megacity: co-occurring contaminants and bacterial community**

Fangfang Ding^a,b,1^, Ye Li^a,1, *^, Tianhao He^a^, Yuyi Wang^a^, Yushan Li^a^, Ye Huang^a^, Guoyu Yin^a^, Jing Yang^a^, Yuyan Liu^c^, Yan Li^d^, Tao Li^e^, Lijun Hou^f^, Min Liu ^a, *^

^a^ Key Laboratory of Geographic Information Science (Ministry of Education), School of Geographic Sciences, East China Normal University, Shanghai 200241, China

^b^ College of Urban and Environmental Sciences, Hubei Normal University, Huangshi 435002, China

^c^ College of Geography and Environmental Science, Hainan Normal University, Haikou 571158, China

^d^ Collaborative Innovation Center of Sustainable Forestry, College of forestry, Nanjing Forestry University, Nanjing 210037, China

^e^ Taihu Basin Shallow Lake Ecosystem Observation and Research Station, Ministry of Water Resources, Wuxi 214024, China

^f^ State Key Laboratory of Estuarine and Coastal Research, East China Normal University, Shanghai 200241, China

**Corresponding authors.*

*E-mail addresses: yli@geo.ecnu.edu.cn (Y. Li), mliu@geo.ecnu.edu.cn (M. Liu)*

^1^ These authors contributed equally to this paper.

**This file includes:**

| Number of pages: | 38 |
| --- | --- |
| Supplementary text: | S1-S13 |
| Number of Tables: | S1-S10 |
| Number of Figures: | S1-S9 |
| References: | 25 |

Supplementary Text

Text S1 The details of the sampling process

At each sampling point, 1 L of overlying water samples were collected in triplicate, stored at 4 °C, and transported to the laboratory for antibiotic testing. A steel water sampler was used to collect 30 L of surface water, and a stainless-steel sieve (300 mesh, pore size 48 μm) was used to filter the surface water in situ. The residue on the sieve was rinsed with deionized water into a 1000 mL wide-mouth brown glass sample bottle to form a concentrated surface water sample. Two parallel samples were collected at each sampling point for microplastic detection. Six sediment mixed samples were collected at each location for antibiotic and microplastic detection, respectively. Each sediment mixed sample consisted of five subsamples, which were collected along a straight line on the river channel parallel to the river edge, with the same distance between each subsample.

Text S2 The details of DNA Extraction and Metagenomic Analysis

1 L water sample was filtered using a 0.22 µm filter membrane, and total DNA was extracted from 0.50 g of sediment and the filtered membrane utilizing the FastDNA Soil Centrifugation Kit (MP Biomedicals, USA), repeated three times. The DNA quality and concentration were checked using a NanoDrop 2000 (Thermo Scientific, USA) and 1% agarose gel electrophoresis (Bio-Rad, USA). The purified DNA was then stored at -80 °C for further analysis. Megahit was used to assemble the best sequences and perform ORF prediction on the results. Prodigal v2.6.3 was used to predict open reading frames (ORFs) for contigs in the splicing results. Genes with a nucleic acid length greater than or equal to 100 bp were selected and translated into amino acid sequences. CD-HIT v4.7 was used to cluster the predicted genes with a clustering similarity of ≥90% to construct a non-redundant gene set and obtain the base sequences of the genes in the non-redundant gene set. Based on the information aligned by SOAPaligner, the abundance information of genes in each sample was counted. Taxonomic and functional annotations of representative sequences of non-redundant gene catalogs were performed using BLASTP against Non-Redundant Protein Sequence Database (NR) and the Comprehensive Antibiotic Research Database (CARD). Species and ARGs abundance analyses were performed based on marker abundance per kilobase per million reads (RPKM).

To further analyze the host bacteria of ARGs in the environment, their potential mobility, and pathogenicity, DeepARG software was employed to annotate the nucleotide sequences of ORFs with ARGs. Based on the ARGs annotation results, contigs carrying ARGs, namely antibiotic resistance contigs (ARCs), were screened. The abundance of ARGs and ARCs was calculated using the bwa-mem algorithm of BWA (https://bio-bwa.sourceforge.net/, version 0.7.17-r1188) using Coverage (×/Gb) units. Species annotation of all ORFs of ARCs was performed using DIAMOND (version 2.0.15.153) based on the NCBI-NR database (version nr_20200306). By predicting the genetic location of ARCs, analyzing MGEs, and annotating species taxonomy, the transmission mechanisms and pathogenicity of ARGs in the environment were explored more deeply.

Text S3 Antibiotics Detected by UPLC-MS.

Surface water samples (1 L) were filtered through a glass fiber filter membrane (GF/F, Whatman), and three parallel samples were set up at each sampling site. The filtrate was acidified to pH 5.0 by dilute hydrochloric acid (1 mol/L), 1 g of Na2EDTA was added to minimize interference with heavy metals in the surface water. Bulk water samples were spiked with surrogate standards to a final concentration of 1.0 ng/mL. The Oasis HLB cartridges (Waters, USA) were sequentially filled with 10 mL of methanol, 10 mL of ultrapure water, and 10 mL of ultrapure water (pH=5), and pre-treated at a flow rate of 1 mL/min. Subsequently, the acid-adjusted filtrate was extracted through a solid phase extraction (SPE) device (Supelco Visiprep system) using pretreated Oasis HLB cartridges. The analytes in the cartridge were eluted with 10 mL of elution solution (methanol: acetonitrile, v/v, 1:1). The eluate was collected and evaporated to 0.5 mL with a gentle stream of nitrogen at 40 ℃. After replacement using 8 mL of methanol, it was again nitrogen-blown to 0.5 mL, transferred to a brown GC vial using dilute formic acid solution to dilute the concentrated eluate to a volume of 1.0 mL, and 20 μL (1 μg/ml) of the internal standard solution was added prior to loading the machine.

Sediment samples were freeze-dried and sieved through a 60-mesh sieve. Three parallel samples were set up for one sample site.1 g of sample was weighed into a glass centrifuge tube (50 mL), and were spiked with 20 ng of surrogate. 1 mL of extraction buffer (0.552 g sodium phosphate dodecahydrate, 0.258 g sodium citrate, 2 g EDTA dissolved in 20 mL of ultrapure water) and 9 mL of acetonitrile were added into a glass centrifuge tube. The mixture was vortexed for 20 min and sonicated for 15 min and centrifuged at 2500 rpm for 5 min after adding 3 g of anhydrous sodium sulfate to separate the solid and liquid phases. The supernatant was pipetted and transferred to a test tube. The same extraction process was repeated twice. Finally, the obtained solution was concentrated, replaced, diluted in the same way and filtered through a 0.45 μm aqueous-phase needle filter. Antibiotic monitoring in SPM also followed the above methodology, using filtered membranes to experimentally derive antibiotic concentrations in SPM.

Text S4 Instrumental analysis

The concentrations of the target antibiotics (Table S1) were determined by high-performance liquid chromatography combined with a tandem triple quadrupole Mass spectrometer (UPLC-MS/MS, Xevo TQ-S, Waters, USA). Briefly, Waters HSS T3 chromatography column (100 mm × 2.1 mm, 1.8 μm) was used to separate the 20 target antibiotics. The mobile phases utilized in this study included ultrapure water containing 0.1% formic acid (v/v), denoted as phase A, and either methanol or acetonitrile, referred to as phase B. These phases were delivered at a flow rate of 0.4 mL/min, with an injection volume of 4 μL. Quantification of all target antibiotics was carried out in the positive electrospray ionization (ESI) mode of MS/MS. Detailed information about the HPLC gradient program for antibiotic separation and the complete MS/MS measurement conditions for individual compounds is presented in Table S2, separately.

Text S5 Detection of target pollutant types

Antibiotics of sulfonamides (SAs), including sulfadiazine (SDZ), sulfapyridine (SPD), sulfamethoxazole (SMX), sulfathiazole (STZ), sulfamerazine (SMR), sulfamethazine (SMZ), and sulfaquinoxaline (SQX), fluoroquinolones (FQs), involving enrofloxacin (EFC), ciprofloxacin (CFC), norfloxacin (NFC), and ofloxacin (OFC), tetracyclines (TCs), including tetracycline (TC), doxycycline (DXC), oxytetracycline (OTC), and chlortetracycline (CTC), macrolides (MLs), containing roxithromycin (RTM) and erythromycin (ETM), chloramphenicols (CPs), including florfenicol (FF), thiamphenicol (TAP), and chloramphenicol (CAP), were detected. The abbreviations of antibiotic concentrations in water phase, sediment phase, and particle phase are prefixed with “W-”, “P-”, and “S-”, respectively.

Text S6 Isolation, Quantification, and Identification of MPs

Microplastics in water samples were extracted using the method with slight modifications [1]. Briefly, concentrated surface water samples were filtered, 0.05 M ferrous sulfate solution was added, and 30% hydrogen peroxide was added to degrade organic matter. The digested solution was filtered onto a 0.45 μm filter membrane (GF/F, 47 mmФ, Whatman) for further monitoring. 200 g sediment sample was weighed and placed in a 1 L glass beaker. Saturated zinc chloride solution was slowly added and mixed thoroughly with a glass rod. After allowing to settle for 5 minutes, saturated zinc chloride solution was continued to be added for flotation. The flotation supernatant solution was transferred to another glass beaker. Repeating the above steps can increase the recovery rate. Subsequently, 25 mL H_2_O_2_ (30%) solution was added to the supernatant, mixed thoroughly with a glass rod, heated to 75 °C in a water bath, left to stand for 24 h, and filtered using a vacuum filtration pump. The filter membrane was placed in a glass culture dish and air-dried in the dark.

Microplastic samples on glass fiber filters were photographed using a stereo microscope (SZX2-ZB16/E3ISPM, OLYMPUS, Japan). Visual identification and counting of microplastics were performed based on their morphological characteristics. Microscope CCD lenses were used to take photos, and the particle size of microplastics was measured using relevant image software, and the length was calculated based on their longest side. A microscopic Fourier transform infrared spectrometer (Spotlight 400, Perkin Elmer, USA) was used to select suspected microplastic particles for chemical composition identification. The obtained spectra had a match of more than 60% with the standard spectra and could be identified as the corresponding substances. Finally, the abundance of microplastics was counted based on the identification results.

Text S7 Microplastic fragmentation model

Plastic waste in the natural environment will change in size and shape over time. Although the complete degradation of microplastics is extremely slow, the probability of microplastics fragmentation is relatively high. Under various effects (such as mechanical wear, photochemical transformation, chemical action and biodegradation), larger plastic fragments will break and split into secondary microplastics, that is, break into smaller microplastics. With the change of shape type, the temporal evolution of MPs fragmentation leads to the distribution pattern of microplastics of different particle sizes.

Based on conditional probability, a model is established to describe the particle size distribution and fragmentation characteristics of MPs in the natural environment. Assuming that the fragmentation of MPs is a constant with a size of x, the aging rate is expressed as:

$\lim_{\Delta x\to0} \frac{P(\left.(x\leq X\leq x+\Delta x)\right|X>x)}{\Delta x}=a_{0}$ (1)

Where X is a random variable representing the size of MPs (mm), x and x+Δx represent the smaller size range (mm); a_0_ represents the aging rate (mm^-1^). According to the definition of MP, X is less than 5 mm. Due to the large differences in the morphology and material of MPs, in order to better consider the probability of MPs breakage, it is assumed that MPs breakage is greatly affected by external factors such as natural and human factors, and is less affected by the physical and chemical properties of MPs themselves. The aging rate is further modified and linked to the size of MPs, expressed as:

$\lim_{\Delta x\to0} \frac{P(\left.(x\leq X\leq x+\Delta x)\right|X>x)}{\Delta x}=ax^{b}$ (2)

Where a is a normalization constant and x^b^ represents size-dependent fragmentation.

According to the definition of conditional probability, formula (2) can be expressed as:

$\lim_{\Delta x\to0} \frac{P(x\leq X\leq x+\Delta x)}{P(X>x)\Delta x}=ax^{b}$ (3)

The cumulative distribution function (CDF) is defined as the following equation:

$F(x)=P(X\leq x)$ (4)

Therefore, formula (5) can be converted into:

$\frac{F(x+\Delta x)-F(x)}{(1-F(x))\Delta x}=\frac{F'(x)}{1-F(x)}=ax^{b}$ (5)

where $F'(x)$ is the derivative of the CDF. Assuming F(0) = 0, use parameters $\alpha$ and $\lambda$ to replace b+1 and a/b+1. Then equation (6) can also be transformed into:

$F(x)=1-e^{-a/(b+1)x^{b+1}}(x\geq0)=1-e^{-\lambda x^{a}}(x\geq0)$ (6)

The parameter $\lambda$ determines the relative position of the CDF (i.e., the size range of MPs), while $\alpha$represents the shape of the CDF (i.e., the fragmentation pattern). It is worth noting that higher values of $\alpha$indicate that MPs are relatively more stable if their size is relatively small [2]. In this paper, the fragmentation coefficient and stability of microplastics in the environment are calculated based on the above model (MPs-Size-range, MPs-Fracture-mode, simpson-MPs-particle-size, simpson-MPs-polymer).

Text S8 Quality assurance and quality control (QA/QC)

Target antibiotics were determined by internal standard method. The standard curves of antibiotics containing nine concentrations levels (0.5, 1, 2, 5, 10, 50, 100, 250, 500 μg/L) were calibrated, with linear correlation coefficients R^2^ > 0.99. Method blanks were used to detect contamination caused by anthropogenic or environmental factors during experiments. The limits of detection (LODs) and limit of quantification (LOQs) of the antibiotics in water were 0.02-0.35 ng/L and 0.05-1.05 ng/L, respectively. In the sediment, the LODs and LOQs were 0.02-0.6 ng/g and 0.04-1.35 ng/g, respectively. In the SPM, the LODs and LOQs were 0.02-1.09 ng/g and 0.03-1.55 ng/g, respectively. The recoveries of the target antibiotics in different environmental media were determined using the standard addition method. The recoveries of these spiked antibiotics in the water, sediment, and SPM samples were 72.1%-125.4%, 60.3%-129.5% and 63.1%-131.3%, respectively. The recovery rates of surrogate standards for different environmental media samples were 63.2%-131.5% and met the assay criteria.

All containers and apparatus were washed thoroughly with filtered ultrapure water before use. To reduce the pollution of airborne microplastics in the field, samples and equipment were covered with glass lids or aluminum foils. Nitrile gloves and a cotton lab gown were worn during field sampling and laboratory operation. All liquid solutions were filtered with 0.45 μm filters before use. The number of microplastics detected in the procedural controls was 0.25 ± 0.32 items per filter, indicating negligible pollution during the analysis process. The recovery rate of microplastics detected during the experiment was 67%-93%.

Text S9 Ecological risk and resistance development risk assessment of antibiotics

Risk quotient (RQ) was used to assess the potential environmental risks of antibiotics. As ecotoxicity from exposure to antibiotic mixtures varied among species, the species that was most sensitive to antibiotics was chosen to calculate the total risk of multiple antibiotics. The total risk quotient (TRQ) of the 20 antibiotics for most sensitive trophic levels was derived from equation (7) [3-5]:

$TRQ=\sum_{i=1}^{n} \frac{MEC_{i}}{PNEC_{i}}=\sum_{i=1}^{n} \frac{MEC_{i}}{min(EC50_{a\lg ae},EC50_{crustaceans},EC50_{fish})_{i}\times(1/AF_{i})}$ (7)

where MEC represents the measured concentration of antibiotics in water; PNEC represents the predicted no-effect concentration of antibiotics in water (Table S7).

Due to the very limited studies on toxicity assessment of antibiotics in sediments, the solid-liquid partition coefficients (*K*_d_) calculated in this study were used to calibrate the PNEC_sediment_ (equation (8))[6]:

$PNEC_{se\dim ent}=PNEC_{water}\times K_{d}$ (8)

where PNEC_sediment_ indicates the predicted no-effect concentration in sediment; PNEC_water_ indicates the predicted no-effect concentration in water.

Antibiotic resistance risk quotient (RRQ) was obtained as the proportion of measured concentration and resistance corresponding to the predicted no-effect concentration in water (PNEC_R_) to assess the resistance development risk (Table S8) [7].

$RRQ\text{=}\frac{MEC}{PNEC_{R}}$ (9)

Estimated daily intake (EDI) of antibiotics from water were calculated, in accordance with the following equation (10):

$EDI\text{=}\frac{\sum_{i} (C_{i}\times\text{IR})}{BW}$ (10)

where *C_i_* (ng/L) is the mean concentration of antibiotics in water; IR (g/day) is the daily consumption rate of exposure medium *i*; and BW (kg) is body weight. According to local statistical yearbooks, information on weight, drinking water data was obtained (Table S9).

Minimum selective concentrations (MSCs) should be used by calculating the acceptable daily intakes (ADIs) and finally assessing the risk of resistance selection due to daily ingestion of antibiotic (Table S10) [8]. Because of the absence of available MSC data from human microbial communities, MSC values were predicted from minimum inhibitory concentrations (MICs) [9]. The microbiological ADI was calculated as the following equation (11):

$ADI_{Mic}=\frac{MIC_{50}\times MCC}{10\times FA\times BW}$ (11)

where MIC50 is the minimum inhibitory concentration of 50% of strains of the most-sensitive human commensal microflora; MCC is the mass of colonic content (150-220 g/day); FA is the available fraction of the oral dose to the colonic microflora (12-20%); and BW is body weight (61 kg) [8,10].

The hazard quotient (HQ) was defined as the health risk of antibiotics and was calculated by dividing the estimated daily intake (EDI, μg/kg/day) by the ADI (μg/kg/day) [11,12]. To assess the effects of a mixture, the Hazard Index (HI) method was the sum of the exposures to individual compounds. HQ or HI values greater than 1 indicated potential risks of adverse health effects. The equation (12) was as follows:

$HI=\sum HQ=\sum\frac{EDI}{ADI}$ (12)

Text S10 Risk assessment of MPs

The hazard index (HI) considered the toxicity of each polymer. The calculation of HI was shown in equation (13):

$HI=\sum S_{n}\times P_{n}$ (13)

Where HI is the microplastic hazard index, S_n_ is the polymer type hazard score [13], P_n_ is the percentage of a specific polymer type [14].

The integrated pollution load index (PLI) was originally proposed for heavy metals. The microplastics assessment model was defined as follows equation (14,15):

$CF_{i}=C_{i}/C_{0i}$ (14)

$PLI_{i}=\sqrt{CF_{i}}$ (15)

Where CF_i_ is the microplastic pollution coefficient relative to the baseline microplastics. C_i_ is the microplastic abundance. C_0i_ is the benchmark for microplastic abundance. PLI_i_ is the microplastic pollution load.

The potential ecological risk index (PERI) can be calculated using equations (16):

$PERI=\sum_{i=1}^{n} E_{i}=\sum_{i=1}^{n} CF_{i}\times T_{i}$ (16)

Where E_i_ represents the potential ecological hazard of polymer i, and T_i_ represents the chemical toxicity coefficient of the polymer [13]. PERI represents the potential ecological hazard of the bound polymer in the sample.

Text S11 Health risk assessment of ARGs

The health risks of ARGs at different sites were evaluated based on four important indicators, namely human accessibility (HA, the ability of ARGs to transfer from the environment to humans), mobility (MO, the ability of ARGs to transfer through horizontal gene transfer), human pathogenicity (HP, the ability of ARGs to transfer from non-pathogenic bacteria to pathogenic bacteria) and clinical availability (CA, the consumption of commonly used antibiotics related to ARGs in clinical practice), combined with gene abundance calculated from metagenomic data [15,16]. The calculation methods of these indicators were as follows:

$HA=Average\text{ }abundance_{human}\times Prevalence_{human}$ (17)

$HP=Number_{pathogenic}\times Number_{all}$ (18)

$CA=\sum_{i=1}^{n} Usage\text{ }of\text{ }antibiotic_{i}$ (19)

Among them, *Average abundance_human_* and *Prevalence_human_* are the average abundance and prevalence of ARGs in human- associated habitats, respectively. *Number_pathogenic_* and *Number_all_* are the number of pathogenic hosts and total hosts of ARGs, respectively. The “n” in CA represents the number of antibiotics to which ARGs are resistant. In addition, the MO of ARGs was determined according to the number of related MGEs in the metagenome. Considering that the health risk index (RI) is proportional to each indicator, the RI of each ARG to human health was obtained by multiplying these indicators:

$RI=HA\times MO\times HP\times CA$ (20)

The RI values of ARGs at different points are calculated as:

$RI_{sample}=\sum_{i=1}^{n} Abundance_{i}\times RI_{i}$ (21)

Where *abundance_i_* and *RI_i_* are the abundance and RI value of each type of ARG_i_ in the sample, respectively.

Text S12 Diversity and relative abundance of ARGs in different environmental media

To analyze the abundance and diversity of ARGs, non-redundant genes identified from the 13 sampling sites were annotated using the CARD database. Simpsoneven index on ARO name level of ARGs showed significant differences between water and sediment phase (*p* < 0.05) (Figure S2A). A total of 882 ARGs on ARO name level were functional genes shared by water and sediment. Additionally, the unique ARO name in the water and sediment phase included 105 ARGs and 72 ARGs, respectively. A total of 502 and 488 shared ARGs on ARO name level were detected across the water samples and sediment samples, respectively (Figure S2B).

Text S13 Biogeographical Pattern of microorganism

A total of 2126.3 million clean reads were generated from water and sediment samples across 13 sampling sites, yielding 28.43 million non-redundant genes. Based on this data, a non-redundant gene set was constructed and compared with the NR database, enabling species taxonomy annotation and the calculation of species abundance by summing the gene abundances corresponding to each species. The results showed that the Chao index at the genus level indicated significant differences in species diversity between water and sediment (*p* < 0.01) (Figure S5A). The data revealed 6673 genera shared between water and sediment samples, with 826 unique to water and 348 unique to sediments. Water samples from various locations contained 3757 genera, while sediments contained 3212 genera (Figure S5B). The number of unique genera was highest in the water and sediments at site S6. The dominant phylum in water were Proteobacteria, Actinobacteria, Bacteroidota, Uroviricota, and unclassified_d_Bacteria, accounting for 41.5%, 19.3%, 12.2%, 7.7%, and 4.3% of total species abundance, respectively. In sediments, the dominant phyla were Proteobacteria, Chloroflexi, Bacteroidota, Nitrospirae, and Acidobacteria, comprising 51.9%, 9.5%, 5.9%, 5.0%, and 4.6% of total species abundance, respectively (Figure S5C). PCoA of microbial taxa revealed that microbial communities exhibited medium-specific heterogeneity and geographical clustering, with significant differences in microbial community distribution patterns between different media, such as urban rivers (W12) and estuaries (W6, S6) (Figure S5D). In W6, W12, and S9, Proteobacteria was the dominant phylum (Figure S5E-F). Water communities were more influenced by stochastic processes and less by deterministic processes. In comparison to sediments, species dispersal in water communities was more restricted (Figure S7A-B).

Table S1 Physicochemical properties of antibiotics.

| Class | Antibiotic | Abbrevia-tion | Molecular  formula | CAS No. | Molecular | Log *K*ow ^a,b^ | p*K*a | Utilization |
| --- | --- | --- | --- | --- | --- | --- | --- | --- |
|  |  |  |  |  | weight (g/mol) |  |  |  |
| SAs | Sulfadiazine | SDZ | C_10_H_10_N_4_O_2_S | 68-35-9 | 250.3 | -0.09 | 2.00, 6.48 | human and veterinary, aquaculture |
|  | Sulfapyridine | SPD | C_11_H_11_N_3_O_2_S | 144-83-2 | 249.3 | 0.35 | 2.90, 8.54 | human |
|  | Sulfamethoxazole | SMX | C_10_H_11_N_3_O_3_S | 723-46-6 | 253.3 | 0.89 | 1.85, 5.60 | human and veterinary, aquaculture |
|  | Sulfathiazole | STZ | C_9_H_9_N_3_O_2_S_2_ | 72-14-0 | 255.3 | 0.05 | 2.20, 7.24 | human and veterinary |
|  | Sulfamerazine | SMR | C_11_H_12_N_4_O_2_S | 127-79-7 | 264.3 | 0.14 | 2.06, 6.90 | human and veterinary |
|  | Sulfamethazine | SMZ | C_12_H_14_N_4_O_2_S | 57-68-1 | 278.3 | 0.89 | 2.65, 7.65 | human and veterinary, aquaculture |
|  | Sulfaquinoxaline | SQX | C_14_H_12_N_4_O_2_S | 59-40-5 | 300.4 | 1.68 | 1.86, 5.56 | veterinary |
| FQs | Ciprofloxacin | CFC | C_17_H_18_FN_3_O_3_ | 85721-33-1 | 331.3 | 0.28 | 3.10, 6.14, 8.70, 10.58 | human and veterinary, aquaculture |
|  | Enrofloxacin | EFC | C_19_H_22_FN_3_O_3_ | 93106-60-6 | 359.4 | 0.7 | 3.86, 6.19, 7.59, 9.86 | veterinary, aquaculture |
|  | Norfloxacin | NFC | C_16_H_18_FN_3_O_3_ | 70458-96-7 | 319.3 | -1.03 | 3.11, 6.10, 8.60, 10.56 | human and veterinary, aquaculture |
|  | Ofloxacin | OFC | C_18_H_20_FN_3_O_4_ | 82419-36-1 | 361.4 | -0.39 | 5.97, 8.28 | human and veterinary |
| TCs | Tetracycline | TC | C_22_H_24_N_2_O_8_ | 60-54-8 | 444.4 | -1.3 | 3.30, 7.68, 9.69 | human and veterinary |
|  | Doxycycline | DXC | C_22_H_24_N_2_O_8_ | 564-25-0 | 444.4 | -0.02 | 7.75 ^c^ | human and veterinary, aquaculture |
|  | Oxytetracycline | OTC | C_22_H_24_N_2_O_9_ | 79-57-2 | 460.4 | -0.9 | 3.27, 7.32, 9.11 | human and veterinary |
|  | Chlorotetracycline | CTC | C_22_H_23_ClN_2_O_8_ | 57-62-5 | 478.9 | -0.62 | 3.30, 7.55, 9.15 | human and veterinary |
| MLs | Erythromycin | ETM | C_37_H_67_NO_13_ | 114-07-8 | 733.9 | 3.06 | 8.90 | human and veterinary |
|  | Roxithromycin | RTM | C_41_H_76_N_2_O_15_ | 80214-83-1 | 837 | 2.75 | 9.17 | human |
| CPs | Florfenicol | FF | C_12_H_14_C_l2_FNO_4_S | 73231-34-2 | 358.21 | -0.33 | 6.84, 13.60 | Veterinary, aquaculture |
|  | Thiamphenicol | TAP | C_12_H_15_C_l2_NO_5_S | 15318-45-3 | 356.22 | -0.27 | 7.8 | human and veterinary |
|  | Chloramphenicol | CAP | C_11_H_12_C_l2_N_2_O_5_ | 56-75-7 | 323.13 | 1.14 | 7.59, 13.55 | human |

^a^ NCBI (National Center for Biotechnology Information), United States. ChemIDPlus, http://chem.sis.nlm.nih.gov/chemidplus/ (last access: 27 August 2021).

^b^ NCBI (National Center for Biotechnology Information), United States. PubChem, https://pubchem.ncbi.nlm.nih.gov/ (last access: 27 August 2021).

^c^ TMIC (The Metabolomics Innovation Centre), Canada. Drugbank database Version 5.0, https://www.drugbank.ca/ (last access: 25 February 2022).

Table S2 Optimized instrumental and SRM conditions of target compounds and their products.

| Class | Antibiotic | Abbreviation | Ionization mode | Retention time | Gaze duration | Input voltage | Parent ion | Daughter ion | Daughter ion | Crash voltage | Crash voltage |
| --- | --- | --- | --- | --- | --- | --- | --- | --- | --- | --- | --- |
| SAs | Sulfadiazine | SDZ | ES+ | 1.85 | 0.038 | 40 | 251.1 | 92.1 | 156 | 26 | 14 |
|  | Sulfapyridine | SPD | ES+ | 2.46 | 0.038 | 44 | 250.1 | 92.1 | 156 | 24 | 16 |
|  | Sulfamethoxazole | SMX | ES+ | 5.78 | 0.024 | 25 | 254.1 | 92.1 | 156 | 28 | 16 |
|  | Sulfathiazole | STZ | ES+ | 2.15 | 0.038 | 23 | 256 | 92.1 | 156 | 25 | 15 |
|  | Sulfamerazine | SMR | ES+ | 2.93 | 0.038 | 44 | 265.1 | 92.1 | 156 | 26 | 16 |
|  | Sulfamethazine | SMZ | ES+ | 4.54 | 0.01 | 34 | 279.1 | 92.1 | 186.1 | 30 | 16 |
|  | Sulfaquinoxaline | SQX | ES+ | 8.33 | 0.038 | 42 | 301.1 | 92 | 156 | 30 | 16 |
|  | Sulfamethoxazole-d4 | SMX-d4 | ES+ | 5.72 | 0.024 | 25 | 258.1 | 96.1 | 160 | 28 | 16 |
| FQs | Ciprofloxacin | CFC | ES+ | 6.09 | 0.025 | 32 | 332.2 | 245.1 | 228.1 | 23 | 18 |
|  | Enrofloxacin | EFC | ES+ | 6.51 | 0.024 | 32 | 360.2 | 203.2 | 245.1 | 36 | 26 |
|  | Norfloxacin | NFC | ES+ | 5.65 | 0.03 | 32 | 320.2 | 233.1 | 276.1 | 25 | 20 |
|  | Ofloxacin | OFC | ES+ | 5.32 | 0.025 | 31 | 362 | 261 | 218 | 25 | 36 |
|  | Norfloxacin-d5 | NEC-d5 | ES+ | 5.6 | 0.025 | 27 | 325.1 | 238.1 | 281.1 | 24 | 18 |
| TCs | Tetracycline | TC | ES+ | 5.23 | 0.025 | 22 | 445.2 | 154 | 410.2 | 26 | 20 |
|  | Doxycycline | DXC | ES+ | 8.62 | 0.024 | 25 | 445.2 | 98 | 154 | 42 | 28 |
|  | Oxytetracycline | OTC | ES+ | 5.48 | 0.025 | 22 | 461.2 | 201.1 | 426.2 | 36 | 19 |
|  | Chlorotetracycline | CTC | ES+ | 8.06 | 0.024 | 34 | 479.2 | 154 | 444.2 | 28 | 20 |
|  | Tetracycline-d6 | TC-d6 | ES+ | 6.62 | 0.024 | 27 | 465.1 | 154 | 430.1 | 28 | 25 |
| MLs | Erythromycin | ETM | ES+ | 8.58 | 0.038 | 52 | 734.6 | 83 | 158.1 | 39 | 30 |
|  | Roxithromycin | RTM | ES+ | 8.68 | 0.038 | 54 | 837.6 | 158.1 | 679.5 | 30 | 22 |
|  | Roxithromycin-d7 | RTM-d7 | ES+ | 8.68 | 0.038 | 36 | 844.8 | 158.1 | 686.7 | 36 | 20 |
| CPs | Florfenicol | FF | ES- | 2.39 | 0.038 | 16 | 356 | 185 | 336 | 20 | 10 |
|  | Thiamphenicol | TAP | ES- | 1.65 | 0.038 | 44 | 354 | 185 | 290.1 | 22 | 12 |
|  | Chloramphenicol | CAP | ES- | 2.57 | 0.038 | 25 | 321 | 151.9 | 257.1 | 18 | 12 |
|  | Chloramphenicol-d5 | CAP-d5 | ES- | 2.57 | 0.038 | 25 | 326.1 | 157 | 262.1 | 18 | 12 |

Table S3 Abundance of ARG types in water (RPKM).

| Abundance of ARG  types in water(RPKM) | W1 | W2 | W3 | W6 | W11 | W4 | W8 | W12 | W5 | W7 | W9 | W10 | W13 |
| --- | --- | --- | --- | --- | --- | --- | --- | --- | --- | --- | --- | --- | --- |
|  | Agricultural area | | | | | Industrial area | | | Commercial/residential area | | | | |
| Multidrug | 8808 | 8840 | 10665 | 23783 | 11175 | 14572 | 15173 | 22135 | 11851 | 12242 | 12012 | 11211 | 10538 |
| Macrolides | 3441 | 3816 | 4062 | 7191 | 3834 | 4903 | 4597 | 5481 | 3796 | 4375 | 4156 | 3912 | 4077 |
| Tetracycline | 3346 | 3910 | 3929 | 4793 | 4007 | 4515 | 4430 | 4407 | 3755 | 4391 | 4015 | 3918 | 3972 |
| Glycopeptide | 2057 | 2011 | 2280 | 4050 | 2405 | 2783 | 2807 | 3799 | 2229 | 2370 | 2495 | 2400 | 2249 |
| Peptide | 1796 | 1840 | 2048 | 3053 | 2279 | 2750 | 2516 | 3655 | 2092 | 2292 | 2274 | 2244 | 2106 |
| Aminocoumarin | 1497 | 1816 | 1803 | 2922 | 1729 | 2155 | 2000 | 2253 | 1746 | 2024 | 1802 | 1628 | 1819 |
| Mupirocin | 759 | 827 | 799 | 1065 | 1008 | 951 | 1028 | 847 | 873 | 992 | 973 | 988 | 860 |
| Pleuromutilin | 680 | 730 | 757 | 1027 | 819 | 904 | 987 | 944 | 778 | 878 | 851 | 812 | 762 |
| Beta-lactam | 613 | 525 | 573 | 1174 | 671 | 852 | 838 | 1423 | 635 | 656 | 659 | 658 | 582 |
| Fluoroquinolone | 452 | 540 | 574 | 733 | 572 | 711 | 684 | 634 | 614 | 664 | 622 | 584 | 597 |
| Sulfonamide | 413 | 466 | 438 | 495 | 473 | 491 | 492 | 515 | 418 | 489 | 469 | 445 | 436 |
| Triclosan | 166 | 141 | 200 | 645 | 178 | 331 | 358 | 563 | 219 | 190 | 214 | 179 | 172 |
| Fosfomycin | 166 | 195 | 233 | 161 | 312 | 309 | 307 | 302 | 258 | 303 | 263 | 267 | 245 |
| Diaminopyrimidine | 213 | 181 | 193 | 343 | 263 | 246 | 278 | 349 | 276 | 247 | 270 | 271 | 188 |
| Phenicol | 181 | 196 | 194 | 496 | 171 | 271 | 218 | 307 | 191 | 209 | 214 | 172 | 193 |
| Aminoglycoside | 109 | 106 | 153 | 668 | 178 | 220 | 299 | 407 | 183 | 175 | 207 | 165 | 139 |
| Rifamycin | 166 | 144 | 181 | 387 | 167 | 242 | 242 | 253 | 203 | 209 | 192 | 182 | 167 |
| Bicyclomycin | 33 | 23 | 38 | 346 | 41 | 81 | 75 | 169 | 44 | 45 | 46 | 42 | 38 |
| Elfamycin | 10 | 19 | 17 | 19 | 16 | 23 | 20 | 10 | 14 | 21 | 10 | 17 | 16 |
| Nucleoside | 1 | 1 | 2 | 10 | 0 | 1 | 3 | 1 | 0 | 2 | 1 | 1 | 0 |
| Fusidic acid | 0 | 1 | 2 | 0 | 3 | 1 | 1 | 2 | 1 | 1 | 1 | 1 | 2 |
| Total abundance | 24908 | 26327 | 29139 | 53361 | 30301 | 37314 | 37352 | 48457 | 30175 | 32776 | 31744 | 30099 | 29159 |

Table S4 Abundance of ARG types in sediment (RPKM).

| Abundance of ARG  types in sediment(RPKM) | S1 | S2 | S3 | S6 | S11 | S4 | S8 | S12 | S5 | S7 | S9 | S10 | S13 |
| --- | --- | --- | --- | --- | --- | --- | --- | --- | --- | --- | --- | --- | --- |
|  | Agricultural area | | | | | Industrial area | | | Commercial/residential area | | | | |
| Multidrug | 25956 | 26618 | 24955 | 20675 | 24443 | 25445 | 26247 | 27427 | 24919 | 26775 | 25634 | 24101 | 25644 |
| Macrolides | 7104 | 7870 | 6882 | 7543 | 7917 | 7136 | 7290 | 7636 | 7036 | 7720 | 6540 | 6943 | 7356 |
| Tetracycline | 5820 | 6352 | 5601 | 6136 | 6697 | 5847 | 5641 | 6298 | 5731 | 6177 | 4987 | 5898 | 6434 |
| Glycopeptide | 4444 | 4884 | 4421 | 4405 | 4999 | 4607 | 4491 | 5068 | 4550 | 4721 | 4276 | 4430 | 5132 |
| Peptide | 3085 | 3150 | 3033 | 3080 | 3388 | 3251 | 3144 | 3437 | 3025 | 3328 | 3036 | 3012 | 3131 |
| Aminocoumarin | 2753 | 2977 | 2736 | 2229 | 2814 | 2865 | 2709 | 2923 | 2680 | 3086 | 2393 | 2774 | 2783 |
| Pleuromutilin | 1399 | 1352 | 1395 | 854 | 1168 | 1442 | 1444 | 1455 | 1381 | 1466 | 1314 | 1382 | 1320 |
| Mupirocin | 1184 | 1131 | 1099 | 1355 | 1395 | 1148 | 1181 | 1191 | 1179 | 1255 | 1095 | 1156 | 1223 |
| Beta-lactam | 1012 | 1063 | 1045 | 934 | 1070 | 1092 | 1116 | 1091 | 1081 | 1170 | 1175 | 1039 | 1081 |
| Fluoroquinolone | 842 | 917 | 810 | 900 | 935 | 850 | 777 | 923 | 770 | 810 | 704 | 818 | 891 |
| Aminoglycoside | 564 | 552 | 583 | 636 | 583 | 591 | 701 | 646 | 573 | 628 | 600 | 602 | 530 |
| Triclosan | 681 | 581 | 503 | 295 | 468 | 509 | 697 | 628 | 564 | 654 | 889 | 516 | 468 |
| Rifamycin | 572 | 643 | 527 | 683 | 677 | 551 | 473 | 517 | 530 | 576 | 402 | 582 | 612 |
| Sulfonamide | 457 | 334 | 395 | 358 | 406 | 378 | 482 | 421 | 458 | 485 | 523 | 430 | 423 |
| Fosfomycin | 259 | 269 | 301 | 125 | 252 | 304 | 251 | 237 | 265 | 241 | 264 | 255 | 268 |
| Phenicol | 226 | 220 | 241 | 284 | 241 | 231 | 235 | 218 | 212 | 249 | 220 | 206 | 228 |
| Diaminopyrimidine | 197 | 171 | 217 | 155 | 143 | 195 | 221 | 218 | 195 | 216 | 248 | 226 | 156 |
| Bicyclomycin | 72 | 83 | 80 | 52 | 66 | 83 | 81 | 71 | 70 | 79 | 76 | 67 | 64 |
| Elfamycin | 36 | 12 | 20 | 27 | 33 | 32 | 24 | 21 | 19 | 17 | 16 | 8 | 25 |
| Nucleoside | 5 | 17 | 15 | 19 | 15 | 10 | 11 | 11 | 16 | 13 | 3 | 14 | 17 |
| Fusidic acid | 1 | 1 | 0 | 0 | 3 | 0 | 0 | 2 | 2 | 2 | 0 | 2 | 2 |
| Total abundance | 56668 | 59194 | 54859 | 50749 | 57711 | 56568 | 57216 | 60438 | 55256 | 59668 | 54395 | 54461 | 57786 |

Table S5 Abundance of MGE types in water (Coverage,×Gb).

| Abundance of MGE  types in water (Coverage,×Gb) | W1 | W2 | W3 | W6 | W11 | W4 | W8 | W12 | W5 | W7 | W9 | W10 | W13 |
| --- | --- | --- | --- | --- | --- | --- | --- | --- | --- | --- | --- | --- | --- |
|  | Agricultural area | | | | | Industrial area | | | Commercial/residential area | | | | |
| Integrase | 15.14 | 2.94 | 0.00 | 34.68 | 12.73 | 36.88 | 15.33 | 119.11 | 5.73 | 6.02 | 7.11 | 7.84 | 7.43 |
| Recombinase | 7.21 | 1.88 | 0.00 | 48.05 | 11.36 | 10.22 | 3.13 | 49.60 | 4.39 | 0.64 | 4.01 | 0.00 | 1.87 |
| Transposase | 106.91 | 95.46 | 60.18 | 258.11 | 90.12 | 99.66 | 87.78 | 207.05 | 84.19 | 83.70 | 76.00 | 125.63 | 59.19 |
| Total abundance | 129.26 | 100.28 | 60.18 | 340.85 | 114.21 | 146.77 | 106.24 | 375.76 | 94.32 | 90.36 | 87.12 | 133.47 | 68.49 |

Table S6 Abundance of MGE types in sediment (Coverage,×Gb).

| Abundance of MGE  types in sediment (Coverage,×Gb) | S1 | S2 | S3 | S6 | S11 | S4 | S8 | S12 | S5 | S7 | S9 | S10 | S13 |
| --- | --- | --- | --- | --- | --- | --- | --- | --- | --- | --- | --- | --- | --- |
|  | Agricultural area | | | | | Industrial area | | | Commercial/residential area | | | | |
| Integrase | 2.65 | 0.00 | 1.43 | 0.00 | 0.00 | 0.00 | 6.69 | 0.00 | 2.15 | 1.76 | 4.94 | 0.00 | 0.00 |
| Recombinase | 0.00 | 0.00 | 0.00 | 0.00 | 0.00 | 0.00 | 13.71 | 0.00 | 6.47 | 0.00 | 1.61 | 0.00 | 0.00 |
| Transposase | 18.54 | 4.17 | 62.23 | 22.53 | 3.97 | 29.66 | 61.48 | 36.53 | 37.86 | 63.88 | 105.36 | 32.40 | 5.58 |
| Total abundance | 21.20 | 4.17 | 63.66 | 22.53 | 3.97 | 29.66 | 81.88 | 36.53 | 46.48 | 65.63 | 111.91 | 32.40 | 5.58 |

**Table S7** Predicted no-effect concentration (PNEC) values of antibiotics on aquatic species

| Class | Chemical | Taxonomic group | E(L)C_50_ (mg L^-1^) | N(L)OEC (mg L^-1^) | Toxicity | AF | PNEC (ng L^-1^) | Minimum PNEC (ng L^-1^) | Reference |
| --- | --- | --- | --- | --- | --- | --- | --- | --- | --- |
| CPs | CAP | Algae |  | 2.5 | Chronic | 100 | 25000 | 25000 |  |
|  |  | Invertebrates |  | 100 | Chronic | 50 | 2000000 |  | [3] |
|  |  | Fish | 1828 |  | Acute | 1000 | 1828000 |  |  |
|  | TAP | Algae | 4.16 |  | Acute | 1000 | 4160 | 4160 |  |
|  |  | Invertebrates | 268.54 |  | Acute | 1000 | 268540 |  | a |
|  |  | Fish | 195.25 |  | Acute | 1000 | 195250 |  |  |
|  | FF | Algae |  | 2500 | Chronic | 100 | 25000 | 25000 |  |
|  |  | Invertebrates |  | 4000 | Chronic | 100 | 40000 |  | [17] |
|  |  | Fish |  | 5000 | Chronic | 100 | 50000 |  |  |
| SAs | SDZ | Algae | 0.14 |  | Acute | 1000 | 135 | 135 |  |
|  |  | Invertebrates | 221 |  | Acute | 1000 | 221000 |  | [18] |
|  |  | fish | 1.50 |  | Acute | 1000 | 1500 |  |  |
|  | SPD | Algae | 5.28 |  | Acute | 1000 | 5280 | 5280 |  |
|  |  | Invertebrates | 21.61 |  | Acute | 1000 | 21610 |  | [17] |
|  |  | Fish | 4310.86 |  | Acute | 1000 | 4310858 |  |  |
|  | SMX | Algae | 0.03 |  | Acute | 1000 | 30 | 30 |  |
|  |  | Invertebrates | 15.51 |  | Acute | 1000 | 15510 |  | [18] |
|  |  | Fish | 562.5 |  | Acute | 1000 | 562500 |  |  |
|  | STZ | Algae | 141.37 |  | Acute | 1000 | 141.37 | 141.37 |  |
|  |  | Invertebrates | 3097.88 |  | Acute | 1000 | 3097.88 |  | a |
|  |  | Fish | 2371.10 |  | Acute | 1000 | 2371.1 |  |  |
|  | SMZ | Algae | 0.103 |  | Acute | 1000 | 103 | 103 |  |
|  |  | Invertebrates | 110.7 |  | Acute | 1000 | 110700 |  | a |
|  |  | fish | >100 |  | Acute | 1000 | 100000 |  |  |
|  | SQX | Algae | 16.11 |  | Acute | 1000 | 16110 | 16110 |  |
|  |  | Invertebrates | 152.22 |  | Acute | 1000 | 152220 |  | a |
|  |  | Fish | 149.09 |  | Acute | 1000 | 149090 |  |  |
| FQs | NFC | Algae |  | 0.0016 | Chronic | 100 | 16 | 16 |  |
|  |  | Invertebrates |  | 0.12 | Chronic | 50 | 2400 |  | [3] |
|  |  | Fish |  | 0.0027 | Chronic | 10 | 270 |  |  |
|  | CFC | Algae |  | 0.5 | Chronic | 100 | 5000 | 1100 |  |
|  |  | Invertebrates | 1.1 |  | Acute | 1000 | 1100 |  | [3] |
|  |  | Fish | 155.36 |  | Acute | 1000 | 155360 |  |  |
|  | EFC | Algae | 0.049 |  | Acute | 1000 | 49 | 49 |  |
|  |  | Invertebrates |  | 5 | Chronic | 50 | 100000 |  | [3] |
|  |  | Fish |  | 10 | Chronic | 10 | 1000000 |  |  |
|  | OFC | Algae | 0.021 |  | Acute | 1000 | 21 | 21 |  |
|  |  | Invertebrates | 17.41 |  | Acute | 1000 | 17410 |  | [3] |
|  |  | fish | >1000 |  | Acute | 1000 | 1000000 |  |  |
| TCs | TC | Algae | 1 |  | Acute | 1000 | 1000 | 1000 |  |
|  |  | Invertebrates | 31.85 |  | Acute | 1000 | 31850 |  | [19] |
|  |  | Fish | 7.29 |  | Acute | 1000 | 7290 |  |  |
|  | OTC | Algae | 1.04 |  | Acute | 1000 | 1040 | 1040 |  |
|  |  | Invertebrates | 18.65 |  | Acute | 1000 | 18650 |  | [19] |
|  |  | Fish | 6.92 |  | Acute | 1000 | 6920 |  |  |
|  |  | Algae | 0.8 |  | Acute | 1000 | 800 | 600 |  |
|  | DXC | Invertebrates | 0.6 |  | Acute | 1000 | 600 |  | [18,19] |
|  |  | Fish | 7.58 |  | Acute | 1000 | 758 |  |  |
|  | CTC | Algae | 0.75 |  | Acute | 1000 | 750 | 530 |  |
|  |  | Invertebrates | 0.53 |  | Acute | 1000 | 530 |  | [19] |
|  |  | Fish | 6.19 |  | Acute | 1000 | 6190 |  |  |
| MLs | ETM | Algae | 0.02 |  | Acute | 1000 | 20 | 20 |  |
|  |  | Invertebrates | 0.94 |  | Acute | 1000 | 940 |  | [18] |
|  |  | fish | >1000 |  | Acute | 1000 | 1000000 |  |  |
|  | RTM | Algae | 0.047 |  | Acute | 1000 | 47 | 47 |  |
|  |  | Invertebrates | 7.1 |  | Acute | 1000 | 7100 |  | [18] |
|  |  | fish | 288.3 |  | Acute | 1000 | 288300 |  |  |

^a^ US EPA (US Environmental Protection Agency), Ecological Structure Activity Relationships (ECOSAR) Predictive Model V2.0. https://www.epa.gov/tsca-screening-tools/ecological-structure-activity-relationships-ecosar-predictive-model, 2018 (accessed 1 April 2019).

**Table S8** Resistance selection predicted no-effect concentration (PNECR) values of antibiotics on aquatic species

| Antibiotics | PNECR (ng/L) ^[3,7,20]^ |
| --- | --- |
| CAP | 8000 |
| TAP | 1000 |
| FF | 2000 |
| SDZ | 16000 |
| SPD | 16000 |
| SMX | 16000 |
| STZ | 16000 |
| SMR | 16000 |
| SMZ | 16000 |
| SQX | 16000 |
| SMM | 16000 |
| TMP | 500 |
| NFC | 500 |
| CFC | 64 |
| EFC | 64 |
| OFC | 500 |
| TC | 1000 |
| OTC | 500 |
| DXC | 2000 |
| CTC | 2000 |
| ETM | 1000 |
| RTM | 1000 |
| AZM | 250 |
| CTM | 250 |
| ETM-H_2_O | 1000 |
| TYL | 4000 |
| AMOX | 250 |

**Table S9** The average body weights, DWI for different age groups

| Age groups | Body weight (kg) | DWI (L/d) |
| --- | --- | --- |
| 2-7 age | 16.6 | 0.846 |
| 8-12 age | 36.8 | 1.233 |
| 13-19 age | 51.05 | 1.3985 |
| 20-50 age | 60.925 | 2.3975 |
| 51-65 age | 61.9 | 2.32665 |
| >65 age | 59.4 | 2.22 |

BW, body weight, DWI, the Drinking Water Intake.

Relevant data for DWI, BW and population division according to different age groups, were taken from the recommended values in the ‘Exposure Factor Handbook’ of the Environmental Protection Agency United States.

EPA, U.S. Environmental Protection Agency (US EPA), 2011. Exposure Factors Handbook. Washing (DC). Available from: http://www.epa.gov/ncea/efh/pdfs/efh-complete.pdf.

**Table S10** Microbiological acceptable daily intake (ADI)

| Antibiotics | Microbiological ADI | | |
| --- | --- | --- | --- |
|  | MIC_50_ | ADI | Support Materials |
|  | (μg/mL) | (μg/kg-bw/day) |  |
| DXC | 2 (*Peptostreptococcus*) | 3.6 | Antimicrobe. Toxicity informaiton for tetracycline. Available from: http://www.antimicrobe.org/d32tab.htm. |
| CTC | N.A. | N.A. |  |
| OTC | 2 (*Lactobacillus* spp.) | 3.6 | Leeuwen, M.F.A.W.J.E.M.v.K.-V.F.X.R.v. Toxicity data for Chlortetracycline and tetracycline. Available from: http://www.inchem.org/documents/jecfa/jecmono/v36je06.htm. |
| TC | 2 (*Peptostreptococcus* spp.) | 3.6 | Leeuwen, M.F.A.W.J.E.M.v.K.-V.F.X.R.v. Toxicity data for Chlortetracycline and tetracycline. Available from: http://www.inchem.org/documents/jecfa/jecmono/v36je07.htm. |
| AZM | N.A. | N.A. |  |
| CTM | 0.5 (*Peptostreptococcus* spp. and *Lactobacillus* spp.) | 0.9 | [21] |
| ETM | 0.1 (*Bifidobacterium*) | 0.18 | European Medicine Agency, Erythromycin: Summary Report (2)-Committee for veterinary medical products. 2000; Available from: https://www.ema.europa.eu/en/documents/mrl-report/erythromycin-summary-report-2-committee-veterinary-medicinal-products_en.pdf. |
| RTM | 1 (*Peptostreptococcus* spp.) | 1.8 | [21] |
| TYL | 20 (*Streptococcus*) | 36 | European Medicine Agency, Tylosin: Summary Report (1)-Committee for veterinary medical products. 2002. |
| CFC | 0.008 (*E. coli*) | 0.014 | [22] |
| EFC | 0.062 (*E. coli*) | 0.11 | European Medicine Agency, Enrofloxacin: summary report(1)-Committee for veterinary medicinal products. 1998; Available from: http://www.emea.europa.eu/pdfs/vet/mrls/enro01en.pdf. |
| NFC | 0.05 (*E. coli)*) | 0.09 | [23] |
| OFC | 0.75 (*P. aeruginosa*) | 1.4 | [24] |
| SDZ | N.A. | N.A. |  |
| SMZ | N.A. | N.A. |  |
| SMX | 11 (*Fusobacterium* spp.) | 20 | [25] |
| SQX | N.A. | N.A. |  |
| SPD | N.A. | N.A. |  |
| STZ | N.A. | N.A. |  |
| TMP | 0.3 (*Peptococcus* and Peptostreptococcus spp.) | 0.54 | [25] |
| FF | 0.36 (*Fusobacteria* spp.) | 0.64 | Agency, E.M. Florfenicol: Summary Report (1)-Committee for veterinary medical products. 1996; Available from: https://www.ema.europa.eu/en/documents/mrl-report/florfenicol-summary-report-1-committee-veterinary-medicinal-products_en.pdf. |

Note: Microbiological ADI= (MIC50×MCC)/(10×EF×BW), MIC50: the concentration inhibiting 50% of the most-sensitive human commensal microflora; MCC: the mass of colonic content (220 g), which was based on necropsy data of 17 accident victims reporting a total amount of content of 222 ± 21 g (wet weight); EF: the effective fraction of the oral dose to the colonic microflora (20%); BW is body weight (61kg);

ADI, Acceptable Daily Intake; N.A., not available.


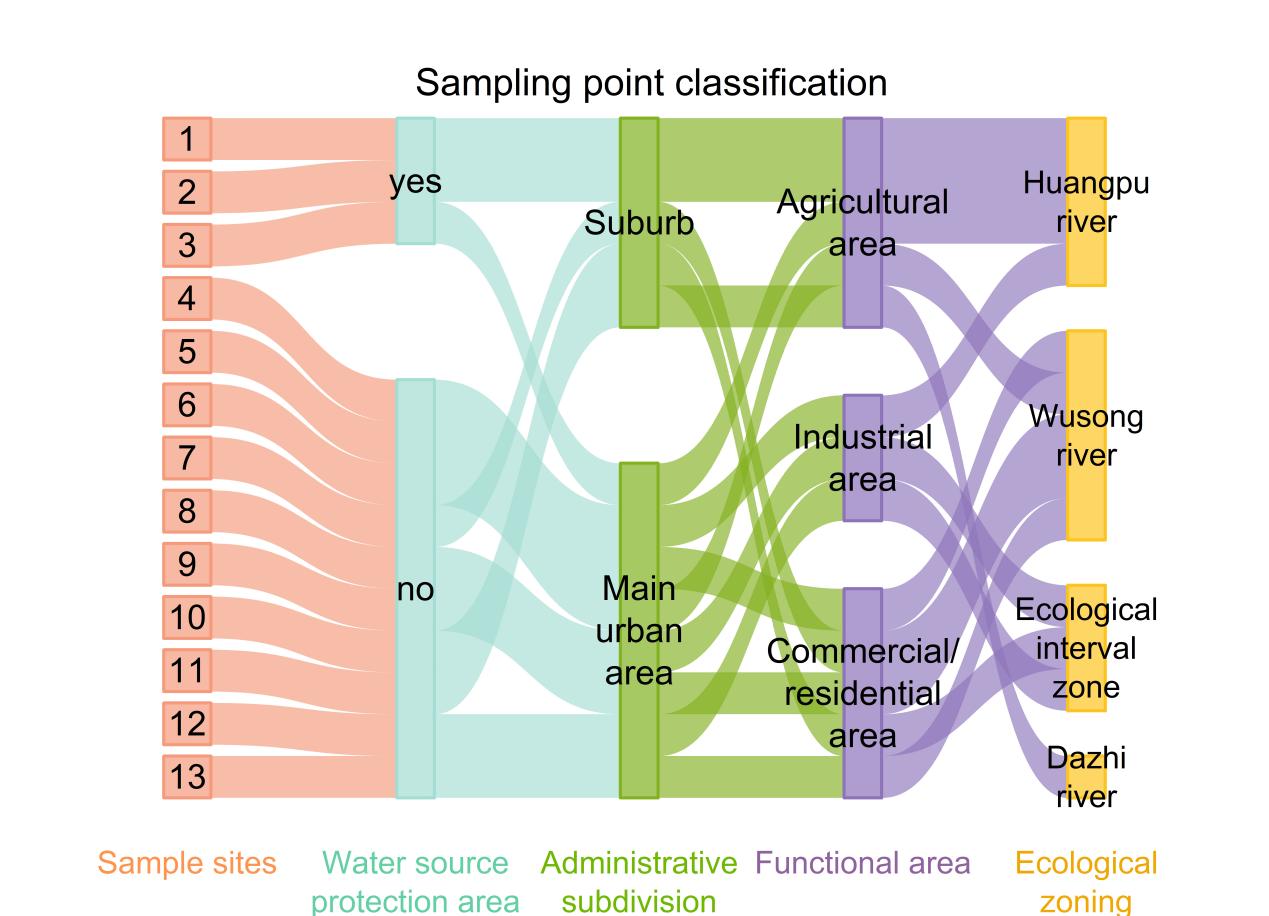


Fig. S1 Sampling points classification.


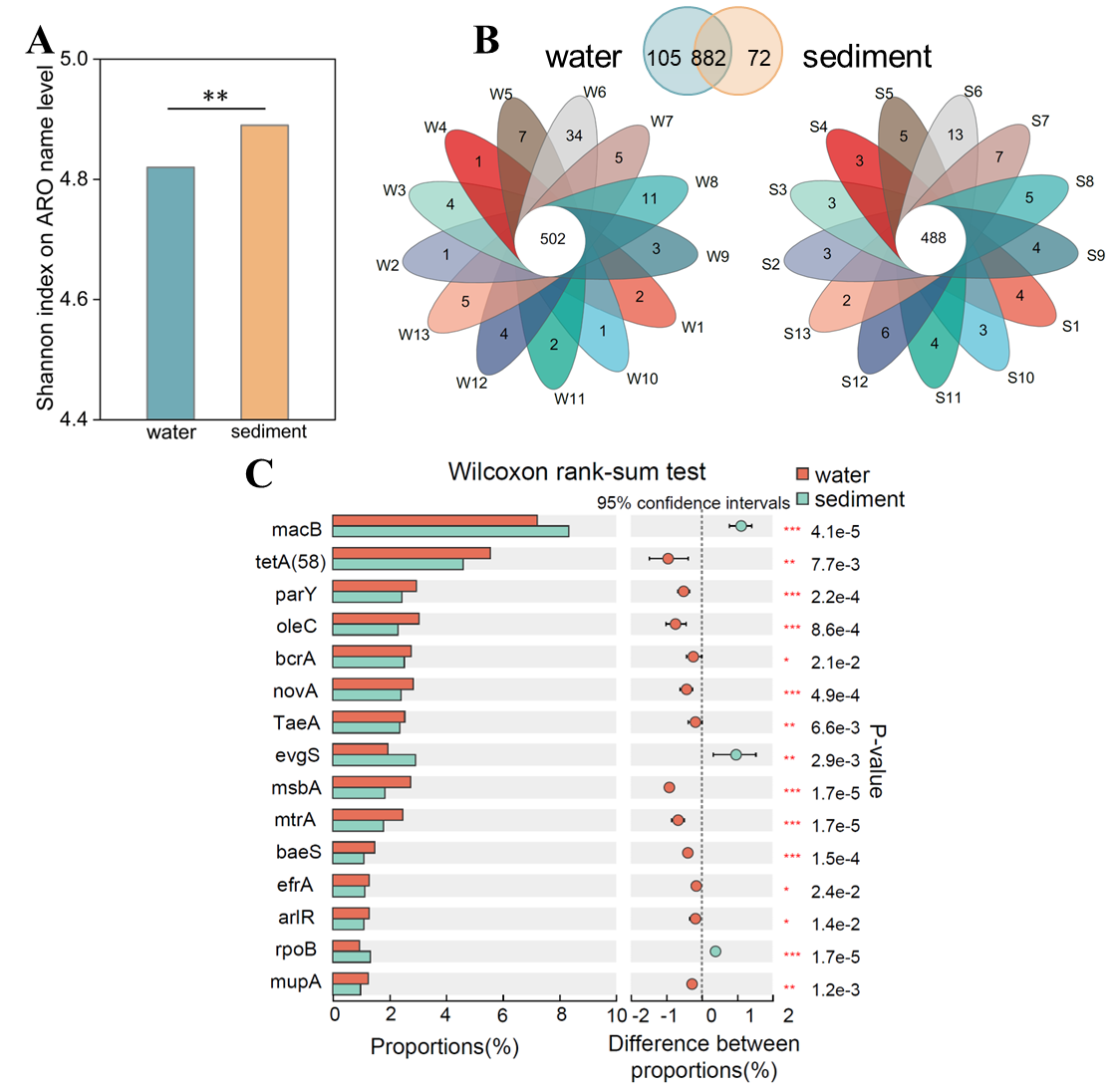


Fig. S2 Diversity and relative abundance of ARGs in different environmental media. (A) Alpha diversity index difference test of ARGs between water and sediment. (B) Analysis of ARGs composition. (C) Differences in relative abundance of ARGs among different groups.


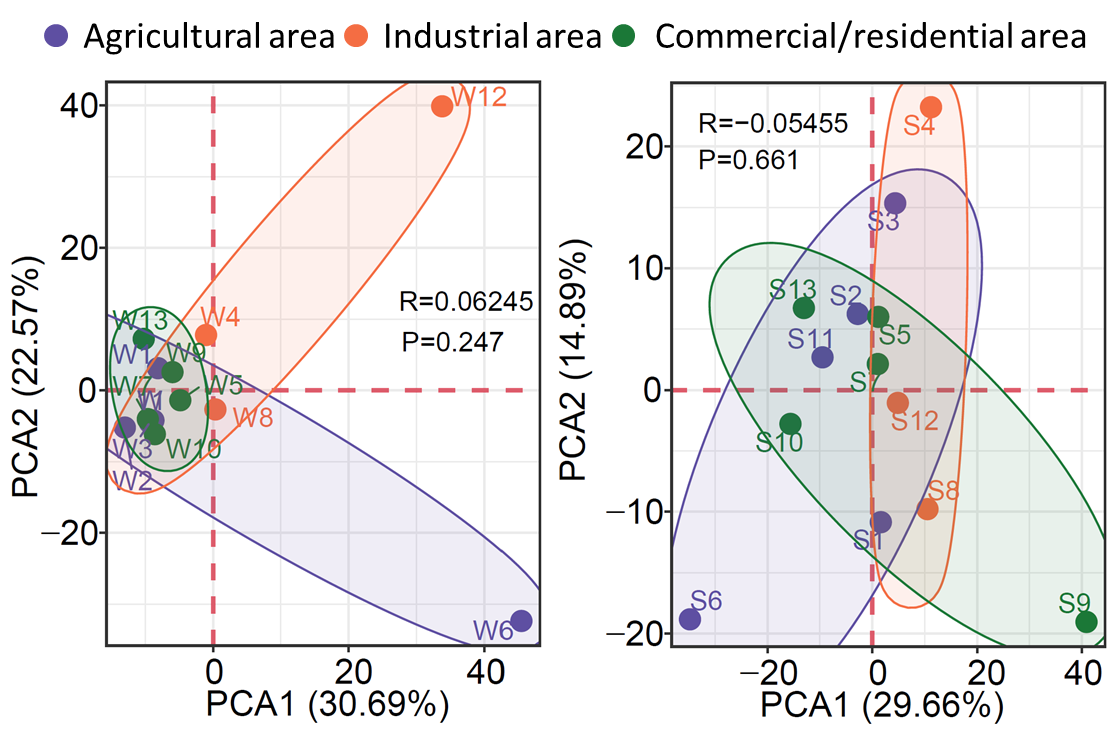


Fig. S3 Principal component analysis (PCA) of ARGs in water (A) and sediment (B).


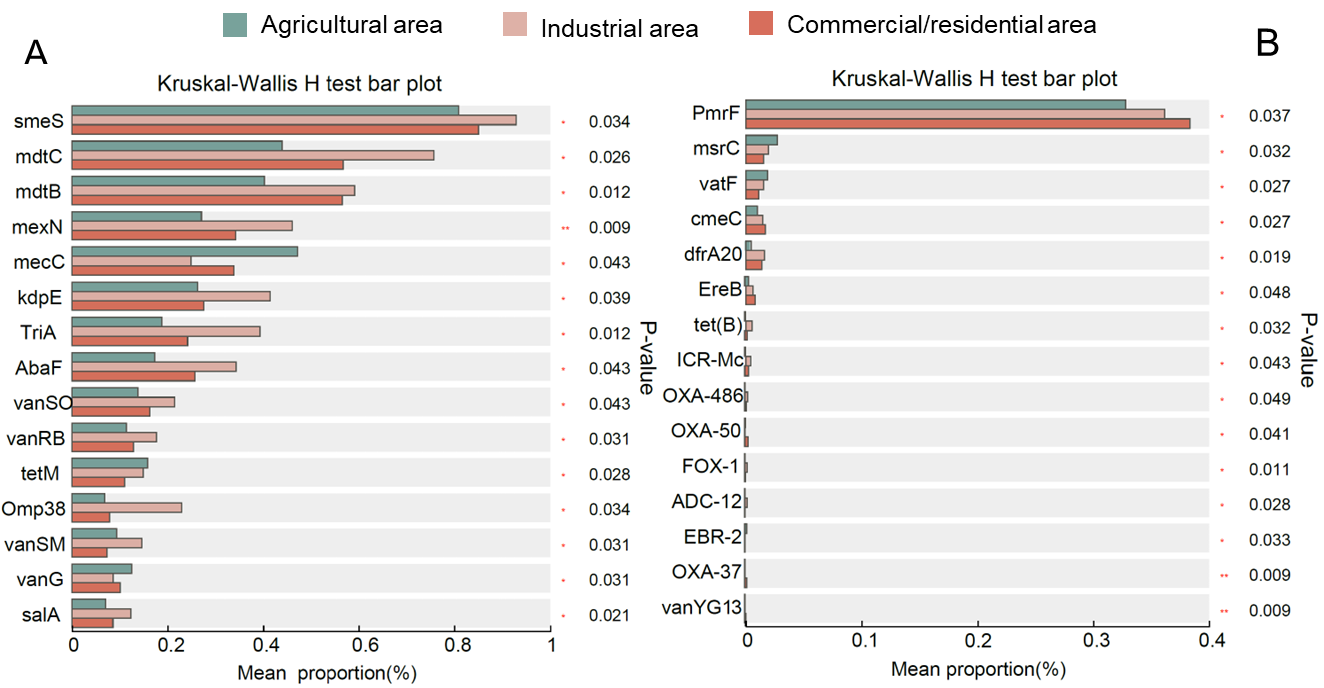


Fig. S4 Differences in relative abundance of ARGs in different functional areas of water (A) and sediment (B).

**
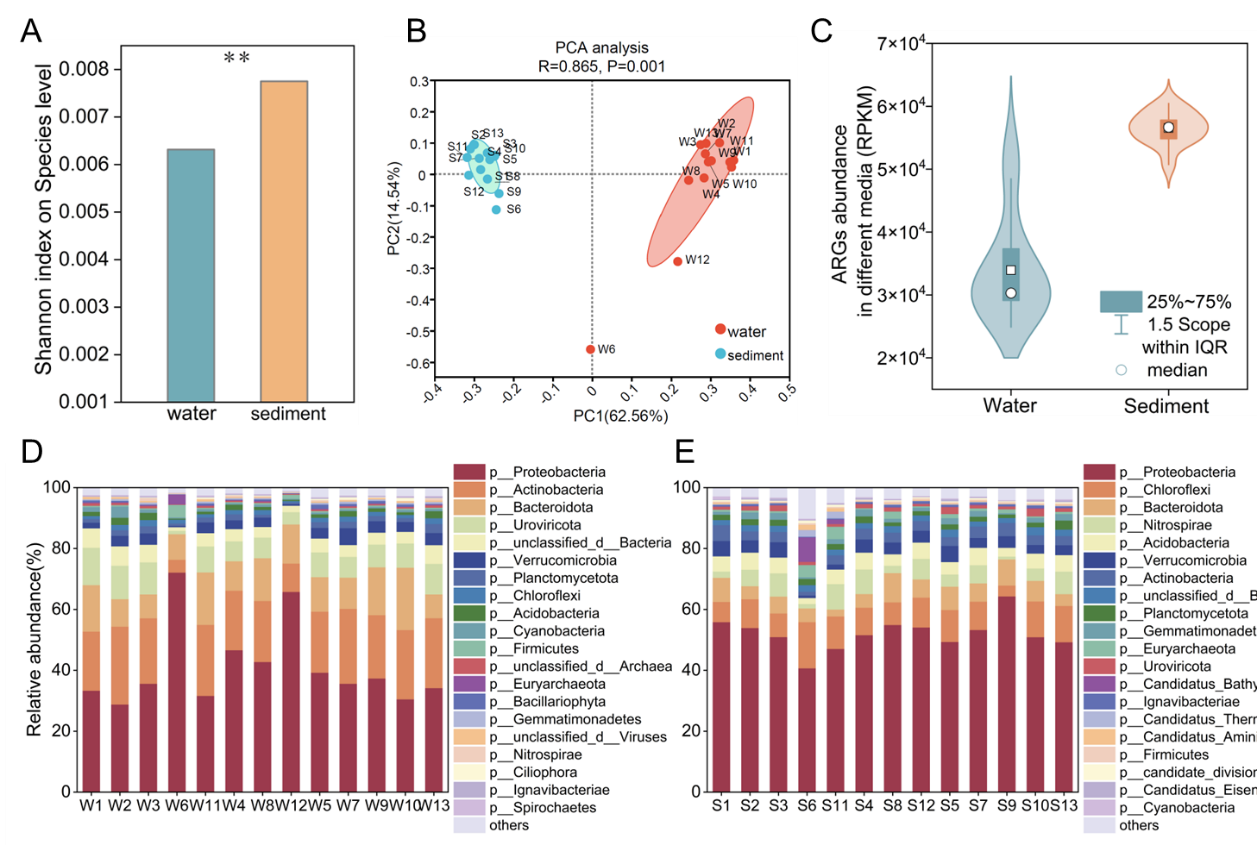
**

Fig. S5 Diversity and abundance of species in different environmental media. (A) Alpha diversity index difference test of species between water and sediment. (B) Principal co-ordinates analysis of species. (C) Differences in abundance of species among different groups. (D) Species composition at the phylum level in water. (E) Species composition at the phylum level in sediment.

**
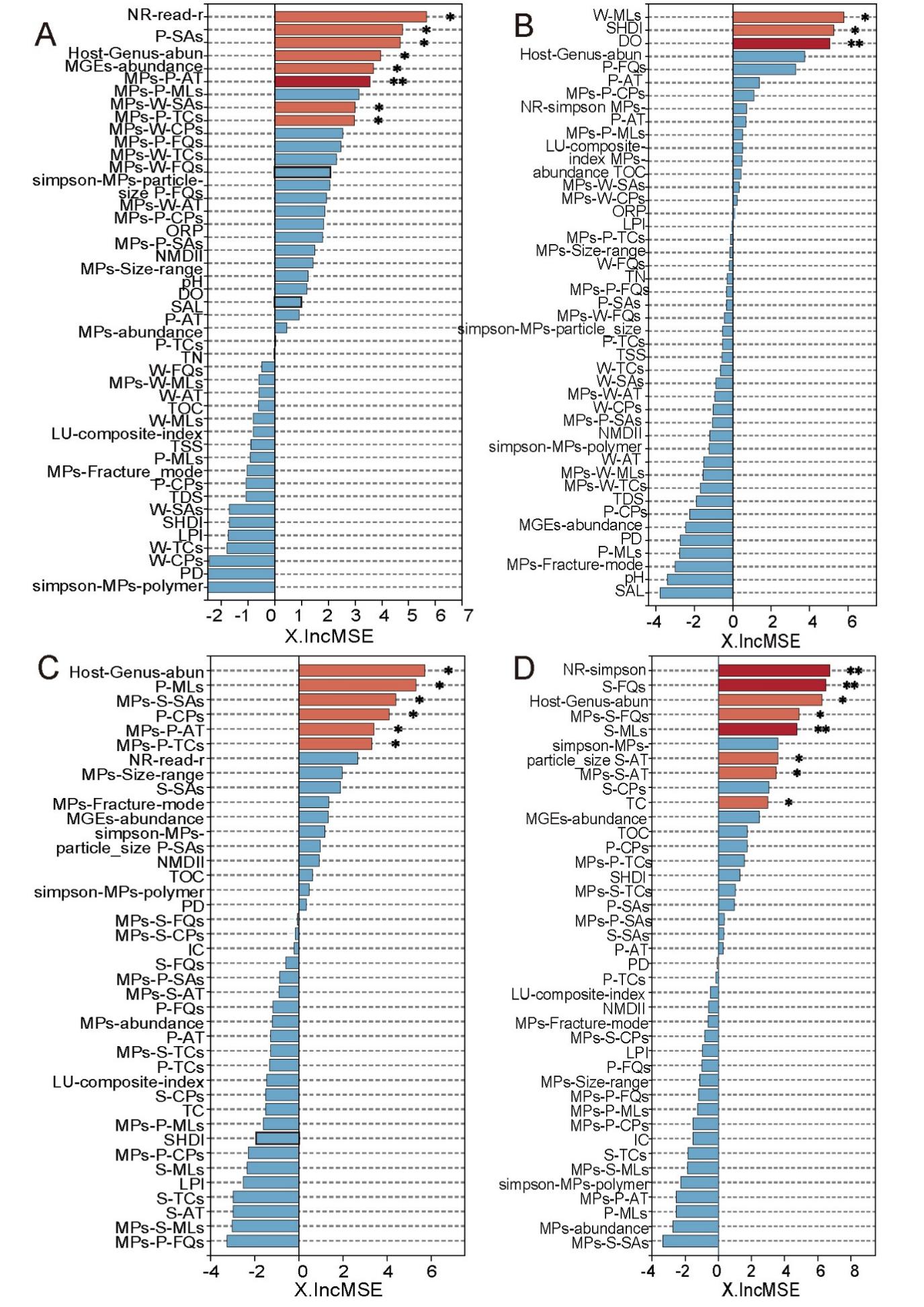
**

Fig. S6 Random forest analysis indicating the effects of natural and human factors on the abundances and diversity of ARGs in water (A-B) and sediment (C-D). * and ** represent significance levels of p < 0.05 and p < 0.01.

Note: “NR-read-r” means relative abundance of species, “Host-Genus-abun” represents host abundance at the genus level, “MPs-P-AT” represents the mass ratio of microplastics to particle-phase antibiotics, and the prefixes “W”, “P”, and “S” correspond to the water phase, particle phase, and sediment phase, respectively. “MPs-Size-range”, “MPs-Fracture-mode” are calculated using the formula in Text11.


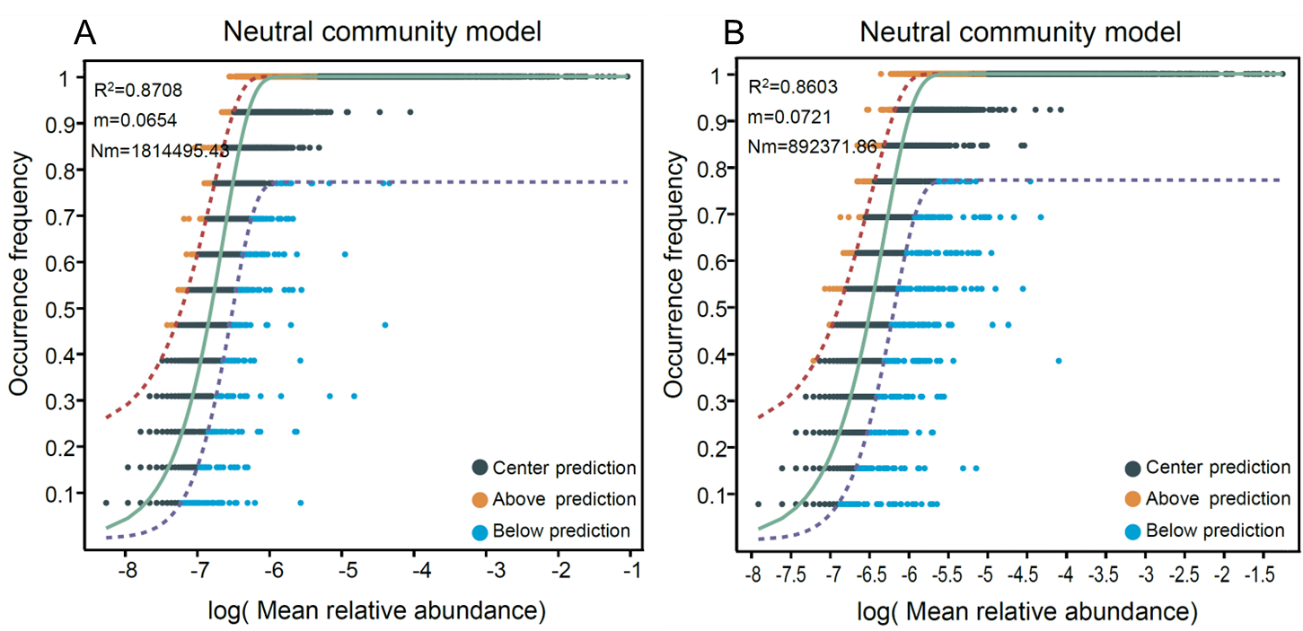


Fig. S7 Analysis of microbial communities in water (A) and sediment (B).


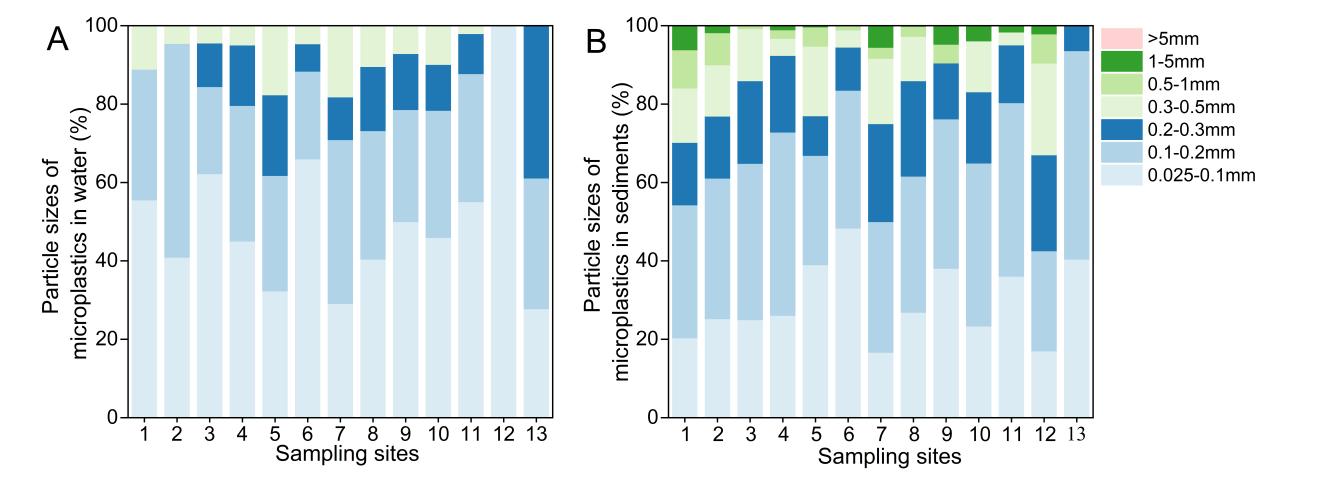


Fig. S8 Particle sizes of microplastic in water (A) and sediment (B).


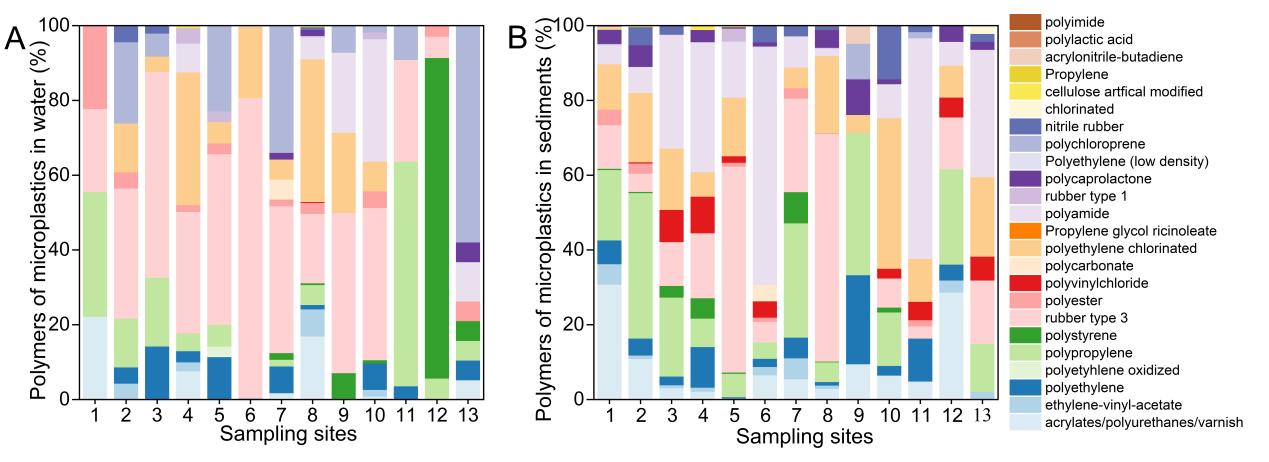


Fig. S9 Polymers of microplastic in water (A) and sediment (B).

**References**

1. W. Yuan, J. Christie-Oleza, E. Xu, J. Li, H. Zhang, W. Wang, L. Lin, W. Zhang, Y. Yang, Environmental fate of microplastics in the world's third-largest river: Basin-wide investigation and microplastic community analysis, Water Res. 210(2022) 118002.
2. S. Lu, C. Lin, K. Lei, M. Xin, X. Gu, M. Lian, B. Wang, X. Liu, W. Ouyang, M. He, Profiling of the spatiotemporal distribution, risks, and prioritization of antibiotics in the waters of Laizhou Bay, northern China, J. Hazard. Mater. 424(2022) 127487.
3. B. Hong, S. Yu, Y. Niu, J. Ding, Q. Lin, X. Lin, W. Hu, Spectrum and environmental risks of residual pharmaceuticals in stream water with emphasis on its relation to epidemic infectious disease and anthropogenic activity in watershed, J. Hazard. Mater. 385(2020) 121594.
4. S. Li, W. Shi, M. You, R. Zhang, Y. Kuang, C. Dang, W. Sun, Y. Zhou, W. Wang, J. Ni, Antibiotics in water and sediments of Danjiangkou Reservoir, China: Spatiotemporal distribution and indicator screening, Environ. Pollut. 246(2019) 435-442.
5. L. Zhang, J. Bai, C. Wang, Z. Wei, Y. Wang, K. Zhang, R. Xiao, M. Jorquera, J. Acuna, M. Campos, Fate and ecological risks of antibiotics in water-sediment systems with cultivated and wild Phragmites australis in a typical Chinese shallow lake, Chemosphere 305(2022) 135370.
6. J. Bengtsson-Palme, D. Larsson, Concentrations of antibiotics predicted to select for resistant bacteria: Proposed limits for environmental regulation, Environ. Int. 86(2016) 140-9.
7. Y. Ben, C. Fu, M. Hu, L. Liu, M. Wong, C. Zheng, Human health risk assessment of antibiotic resistance associated with antibiotic residues in the environment: A review, Environ. Res. 169(2019) 483-493.
8. E. Gullberg, S. Cao, O. Berg, C. Ilback, L. Sandegren, D. Hughes, D. Andersson, Selection of resistant bacteria at very low antibiotic concentrations, PLoS Pathog. 7(2011) e1002158.
9. R. Gretchen M. Bruce, and Shane A. Snyder, Toxicological Relevance of Pharmaceuticals in Drinking Water, Environ. Sci. Technol. (2010) 5619-5626.
10. Y. Zhang, W. Tang, Y. Wang, M. Nian, F. Jiang, J. Zhang, Q. Chen, Environmental antibiotics exposure in school-age children in Shanghai and health risk assessment: A population-based representative investigation, Sci. Total Environ. 824(2022) 153859.
11. Z. Fei, S. Song, J. Gao, Y. Song, X. Xiao, X. Yang, D. Jiang, D. Yang, Antibiotic residues in chicken meat in China: Occurrence and cumulative health risk assessment, J. Food Compos. Anal. 116(2023) 105082.
12. D. Lithner, Å. Larsson, G. Dave, Environmental and health hazard ranking and assessment of plastic polymers based on chemical composition, Sci. Total Environ. 409(2011) 3309-3324.
13. L. Yang, S. Kang, X. Luo, Z. Wang, Microplastics in drinking water: A review on methods, occurrence, sources, and potential risks assessment, Environ. Pollut. 348(2024) 123857.
14. Z. Zhang, Q. Zhang, T. Wang, N. Xu, T. Lu, W. Hong, J. Penuelas, M. Gillings, M. Wang, W. Gao, H. Qian, Assessment of global health risk of antibiotic resistance genes, Nat. Commun. 13(2022) 1553.
15. L. Zhu, R. Li, K. Yang, F. Xu, C. Lin, Q. Chen, D. Zhu, Q. Sun, Y. Zhu, L. Cui, Quantifying health risks of plastisphere antibiotic resistome and deciphering driving mechanisms in an urbanizing watershed, Water Res. 245(2023) 120574.
16. L. Wang, P. Li, Q. Zhang, W. Wu, J. Luo, D. Hou, Modeling the Conditional Fragmentation-Induced Microplastic Distribution, Environ. Sci. Technol. 55(2021) 6012-6021.
17. J. Du, H. Zhao, Y. Wang, H. Xie, M. Zhu, J. Chen, Presence and environmental risk assessment of selected antibiotics in coastal water adjacent to mariculture areas in the Bohai Sea, Ecotoxicol. Environ. Saf. 177(2019) 117-123.
18. W. Li, L. Gao, Y. Shi, J. Liu, Y. Cai, Occurrence, distribution and risks of antibiotics in urban surface water in Beijing, China, Environ. Sci. Proc. Imp. 17(2015) 1611-1619.
19. S. Li, W. Shi, W. Liu, H. Li, W. Zhang, J. Hu, Y. Ke, W. Sun, J. Ni, A duodecennial national synthesis of antibiotics in China's major rivers and seas (2005-2016), Sci. Total Environ. 615(2018) 906-917.
20. X. Zhang, J. Zhang, Q. Han, X. Wang, S. Wang, X. Yuan, B. Zhang, S. Zhao, Antibiotics in mariculture organisms of different growth stages: Tissue-specific bioaccumulation and influencing factors, Environ. Pollut. 288(2021) 117715.
21. D. Citron, M. Appleman, Comparative in vitro activities of ABT-773 against 362 clinical isolates of anaerobic bacteria, Antimicrob. Agents Ch. 45(2001) 345-8.
22. S. Jeong, Y. Song, J. Cho, Risk assessment of ciprofloxacin, flavomycin, olaquindox and colistin sulfate based on microbiological impact on human gut biota, Regul. Toxicol. Pharm. 53(2009) 209-16.
23. N. Chin, H. Neu. In vitro activity of enoxacin, a quinolone carboxylic acid, compared with those of norfloxacin, new beta-lactams, aminoglycosides, and trimethoprim, Antimicrob. Agents Chemother. 24(1983) 754-763.
24. A. Grillon, F. Schramm, M. Kleinberg, F. Jehl, Comparative Activity of Ciprofloxacin, Levofloxacin and Moxifloxacin against Klebsiella pneumoniae, Pseudomonas aeruginosa and Stenotrophomonas maltophilia Assessed by Minimum Inhibitory Concentrations and Time-Kill Studies, PLoS One 11(2016) e0156690.
25. J. Wust, T. Wilkins, Susceptibility of anaerobic bacteria to sulfamethoxazole/trimethoprim and routine susceptibility testing, Antimicrob. Agents Chemother. 14(1978) 384-390.
